# A multi-source behavioral data framework for interpretable urban tourism forecasting

**DOI:** 10.1038/s41598-025-32127-2

**Published:** 2025-12-24

**Authors:** Zirui Nie, Zhonghua Nie

**Affiliations:** 1 Graduate School of Urban Environmental Sciences, Tokyo Metropolitan University, Tokyo, 192-0364 Japan; 2 North China University of Technology, No. 5, Jinyuanzhuang Road, Shijingshan District, Beijing, 100143 China

**Keywords:** Tourism forecasting, Multi-source behavioral data, Graph neural network, Deep learning, Emotion analysis, Structural equation modeling, Complex networks, Complex networks, Mathematics and computing, Science, technology and society

## Abstract

Urban tourism demand prediction remains challenging due to its volatility and complex behavioral patterns. To address these issues, a hybrid forecasting framework integrating Long Short-Term Memory (LSTM) networks with Graph Neural Networks (GNNs) was developed. The framework utilizes multi-source behavioral data to improve forecasting accuracy and robustness. The dataset includes social media sentiment, online travel agency (OTA) activity, meteorological information, and mobile signaling records from eight representative Chinese cities collected between 2022 and 2024. The proposed model achieves a Mean Absolute Percentage Error (MAPE) of 6.31% and a trend accuracy of 83.7%, both outperforming single-model benchmarks. Feature interpretability and structural equation modeling (SEM) analyses indicate that sentiment indices, user engagement, and holiday effects are the main determinants of forecasting accuracy. The study offers a scalable and interpretable intelligent forecasting paradigm for smart tourism management, clarifying how data heterogeneity and model adaptability jointly enhance predictive performance in urban tourism contexts.

**Supplementary Information:**

The online version contains supplementary material available at 10.1038/s41598-025-32127-2.

## Introduction

With the growing dynamism, nonlinearity, and diversity of tourist behavior, forecasting urban tourism demand has become a central challenge in the development of smart tourism systems^1^. Traditional models that rely solely on historical data are often inadequate for addressing sudden demand surges caused by holidays, policy changes, or unexpected disruptions^2,3^. In high-frequency and rapidly changing environments, approaches based on a single data source or static modeling framework are unable to represent the intertwined timing and spatial patterns of tourist activity^4^. Meanwhile, destination management organizations (DMOs) and stakeholders such as online travel agencies (OTAs) increasingly emphasize the need for forecasting methods that offer both higher accuracy and stronger interpretability^5,6^.

The rapid expansion of digital platforms and social media has transformed tourism behavior data into a multi-source, high-frequency, and heterogeneous form, encompassing diverse dimensions such as social text, review sentiment, search activity, meteorological factors, transportation records, and mobile signaling information^7,8^. These data sources create a more comprehensive feature space for modeling but also introduce challenges due to differences in structure, sampling frequency, and data quality, making integration and noise control difficult^9^. Many existing studies continue to depend on single-source data, which limits the ability to represent interrelationships among behavior-driven variables^10^. Developing a systematic multi-source data fusion framework has become essential for achieving feature alignment and establishing unified model inputs^11^.

The development of deep learning has introduced new possibilities for spatiotemporal forecasting in tourism research^12,13^. Long short-term memory (LSTM) networks effectively capture periodic and nonlinear patterns within time series data^14^, while graph neural networks (GNNs) are well suited to represent spatial relationships and interconnections among urban areas^15–17^. In the context of tourism, visitor mobility reflects both temporal and spatial dynamics—it evolves over time based on behavioral patterns and is shaped by location and transportation networks^18^. Therefore, integrating LSTM and GNN models provides a complementary framework capable of simultaneously identifying when and where tourism demand occurs^19^.

Recent advances in spatiotemporal graph neural networks (ST-GNNs) have significantly improved predictive learning in urban computing with structured temporal data. Key studies include a comprehensive review of ST-GNN architectures and applications^20^, a dual-view WaveNet model for urban event forecasting^21^, an automated fusion framework for hotspot prediction^22^, an ensemble benchmark model for urban hotspot analysis^23^, and a dilated spatiotemporal graph framework for traffic forecasting^24^. Unlike these studies, which focus mainly on single domains such as transportation or hotspot detection, the present work examines behavior-driven tourism demand characterized by strong variability and high heterogeneity. The framework integrates multiple heterogeneous data sources, including social sentiment, OTA behavioral data, mobile signaling, meteorological factors, and official statistics. The proposed LSTM–GNN hybrid model extends existing ST-GNN approaches in three main aspects: (1) aligning spatiotemporal features and constructing urban graph representations across multiple data sources; (2) implementing weighted and stacked fusion of sequence and graph components; and (3) enhancing interpretability through SHAP/Lasso analysis and validating mechanisms using structural equation modeling (SEM). Overall, the framework clarifies how data complexity and model adaptability jointly influence forecasting accuracy in urban tourism demand analysis.

Beyond model development and comparison, a key challenge lies in the limited understanding of the mechanisms underlying forecasting errors. Forecasting outcomes are shaped by multiple interacting factors, including data quality, feature diversity, model structure, and parameter configuration^6^. In the absence of a clear mechanistic explanation, even models with high forecasting accuracy may lack interpretability and practical value, especially when addressing questions in real-world applications such as why a forecast failed or which variables most strongly influence performance^25,26^. Incorporating error attribution and variable sensitivity analysis into the modeling process is essential. Methods such as SHapley Additive exPlanations (SHAP) and Local Interpretable Model-agnostic Explanations (LIME) can identify influential features and reveal how the model responds to them^27^. Complementary techniques, including stepwise regression, ridge regression, nonlinear fitting, and mediation analysis, can further clarify causal pathways linking multi-source data, model adaptability, and forecasting performance^28^. This exploration advances the theoretical understanding of tourism forecasting and provides a stronger scientific basis for building more adaptive and reliable forecasting systems in practice.

Guided by theoretical motivation and practical demand, the study aims to develop a tourism demand forecasting framework that integrates multi-source big data, compares deep learning models, and clarifies the mechanisms underlying forecasting accuracy. The research pursues three main objectives. The first is to design a systematic approach for multi-source data collection, preprocessing, and integration, including emotion quantification, popularity index construction, and temporal–spatial alignment, to establish a unified data input system. The second is to build LSTM-based, GNN-based, and hybrid models and evaluate their forecasting performance across different temporal and spatial scales and feature combinations. The third is to propose an error analysis and mechanism modeling framework that explains how feature complexity and model adaptability jointly influence forecasting performance, identifying key variables and causal pathways. Methodologically, the study moves beyond single-model dependence by achieving integration at both the data fusion and model architecture levels within tourism demand forecasting. Theoretically, it bridges the gap between model selection, forecasting error, and mechanistic interpretation, offering a more systematic understanding of model behavior. Practically, the framework provides destination management organizations, tourism platforms, and transport authorities with a highly accurate and interpretable forecasting tool to support dynamic decision-making in demand regulation, resource allocation, and risk management. The findings hold broad implications for advancing both academic research and operational practice in smart tourism management.

In summary, the study contributes to tourism demand forecasting in three key areas:

Methodological Contribution: A hybrid forecasting framework combining LSTM and GNN models is proposed to capture both temporal dynamics and spatial dependencies. The incorporation of an adaptive fusion strategy strengthens model robustness and enhances spatiotemporal adaptability.

Data Contribution: A comprehensive multi-source behavioral dataset is developed, encompassing eight major Chinese cities from 2022 to 2024. The dataset integrates social media sentiment, OTA platform activity, meteorological information, and mobile signaling data, providing a detailed representation of the evolving patterns of urban tourism demand.

Interpretability Contribution: An interpretability analysis pipeline is established by integrating SHAP, LIME, and SEM methods to identify how emotional, behavioral, and environmental variables interact to influence forecasting accuracy. The framework offers transparent and theoretically grounded insights to support intelligent decision-making in smart tourism management.

## Materials and methods

### Data source selection and sample space construction

Eight representative Chinese cities (Beijing, Shanghai, Chengdu, Hangzhou, Xi’an, Sanya, Chongqing, and Qingdao) were selected as the research sample, covering the period from January 2022 to December 2024. These cities reflect distinct patterns of tourism development, including northern transportation hubs, cultural destinations in the Yangtze River Delta, and southern coastal resorts. Each city was chosen for its strong data availability, well-developed tourism infrastructure, and significant visitor capacity, ensuring both diversity and representativeness.

**Fig. 1:**
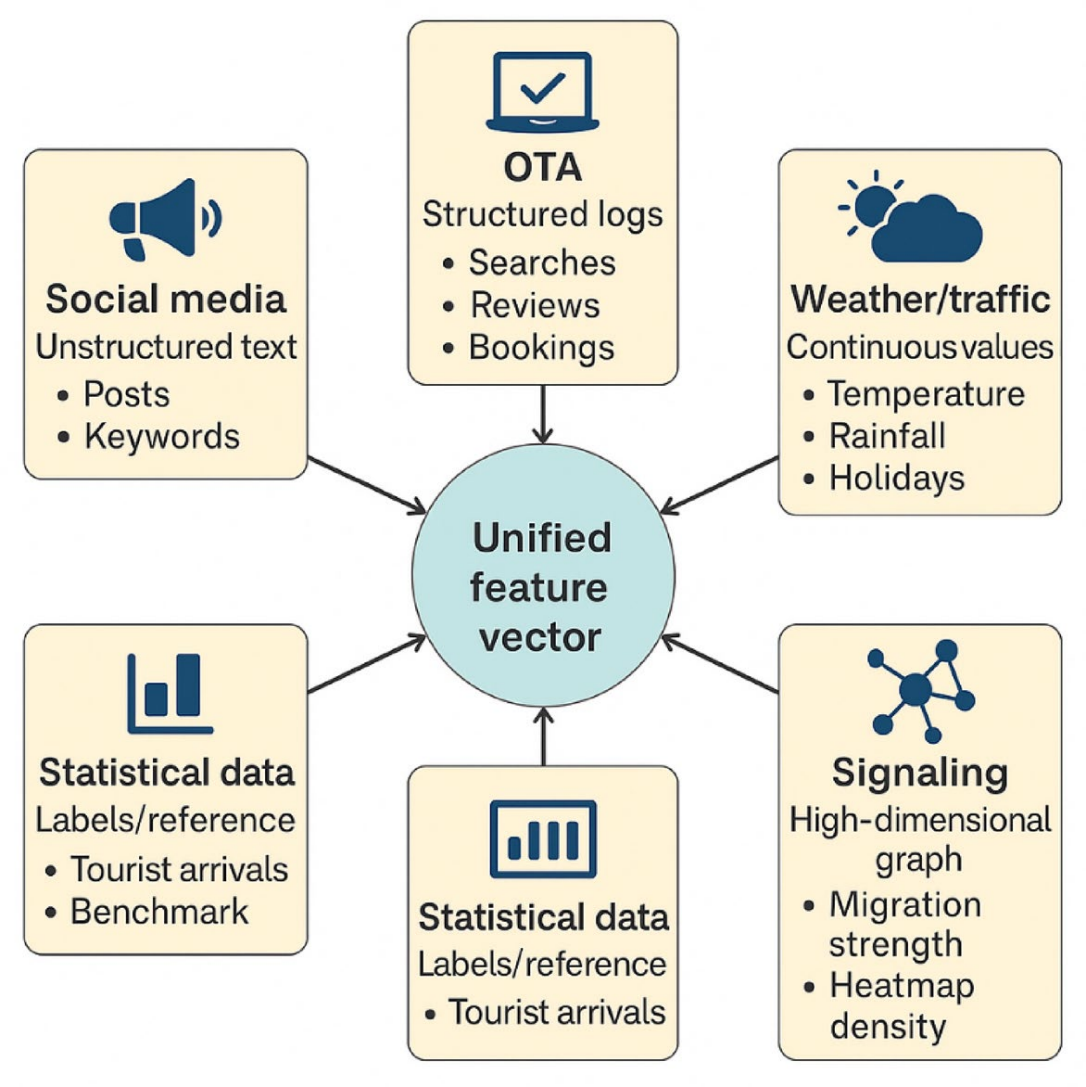
Schematic of the multi-source tourism behavior data structure used in this study. This diagram illustrates the five core categories of data sources comprising the multi-source tourism behavior database, including social media text, OTA platform user behavior, meteorological and traffic information, mobile signaling data, and macro-level tourism statistics. The data sources are categorized along structured/unstructured and static/dynamic dimensions and are temporally and spatially aligned during the data integration phase.

A multi-source tourism behavior database was constructed, comprising five categories of data:

① Social media text data: Public microblog posts containing keywords such as “tourism,” “attractions,” and “travel” were collected through the Sina Weibo API. Metadata, including timestamps, user identifiers, and geolocation coordinates, were extracted, resulting in approximately 12 million valid entries.
② OTA platform behavior data: User search, booking, and review sentiment data were collected from Ctrip and Mafengwo using web crawlers, generating around 3.8 million records.
③ Weather and traffic data: Meteorological data were obtained from the China Meteorological Administration API, including daily temperature, precipitation, and weather conditions, along with public holiday indicators. Traffic data were retrieved from the Amap Open Platform, providing congestion indices and travel conditions near major tourist attractions.
④ Mobile signaling trajectory data: Anonymized signaling data from telecom operators were used to construct daily tourist heatmaps and intercity mobility networks, yielding approximately 6.7 million trajectory records.
⑤ Macro-level tourism statistics: Monthly data on tourist arrivals, tourism revenue, and visitor origin composition were collected from the China Tourism Academy and local cultural and tourism bureaus. These statistics served as supplementary indicators and validation references for the model’s target variable (Fig. 1).

### Data cleaning and standardized processing

To enable joint modeling of multi-source data with consistent timing, scale, and reliability, a unified procedure for missing-value imputation and anomaly correction was applied across all datasets. All records were aggregated at a daily level to match the forecasting interval of the model and the operational frequency required for managerial decision-making. Spatial entities such as place names and tourist attractions were standardized and mapped to the eight study cities, minimizing biases from inconsistent naming and ensuring accurate integration for graph-based modeling. Textual data were processed using a dual-channel sentiment extraction approach that combined a lexicon-based method with a pre-trained language model. This process produced city-level daily sentiment indices and sentiment volatility indicators, reflecting early behavioral signals relevant to tourism activity. The complete data processing workflow and parameter configurations are detailed in Appendix S1.

### Feature engineering and data integration

To capture both short-term variations and longer-term trends in tourism demand, multi-scale temporal features were constructed using 7-, 14-, and 30-day windows. Multi-source variables were grouped into four dimensions (behavioral, environmental, historical, and mobility) to ensure comprehensive coverage and interpretability. The forecasting targets corresponded to city-level daily and weekly tourist arrivals, while input sequences incorporated lagged multi-source data. To enhance comparability and stabilize distributions across data sources, standardization and distributional robustness transformations were applied. The resulting unified temporal–feature tensor provided a consistent input structure for the LSTM, GNN, and hybrid forecasting models. Variable definitions, temporal window design, and transformation procedures are detailed in Appendix S1.

Temporal alignment between the selected windows and the intrinsic periodicity of tourism demand was verified through autocorrelation function (ACF) and fast Fourier transform (FFT) analyses. The results revealed a strong autocorrelation peak at a 7-day lag, indicating a dominant weekly pattern associated with weekend and short-holiday travel. Secondary peaks at 14 and 28–31 days reflected biweekly and monthly periodicities. Based on these findings, the 7-, 14-, and 30-day windows were adopted to represent short-term fluctuations, medium-term dynamics, and long-term inertia. Cross-validation tests showed that extending the window beyond 30 days reduced forecasting accuracy, increasing the root mean square error (RMSE) by less than 1.2%, confirming that the chosen configuration achieved an effective balance between accuracy and computational efficiency. Detailed results are presented in Table 1, supporting the rationality and interpretability of the selected temporal scales.

**Table 1:** Periodicity analysis of city-level tourism demand and rationale for window selection.

City	Dominant periods (days, from ACF/FFT)	Peak correlation/power	Main interpreted cycle	Selected window representation	Validation RMSE change (%)
Beijing	7, 28	0.74 / 0.63	Weekly + monthly seasonality	7-day, 30-day	–
Shanghai	7, 30	0.71 / 0.58	Weekly + monthly	7-day, 30-day	–
Chengdu	7, 14, 29	0.69 / 0.55	Weekly + biweekly + monthly	7-, 14-, 30-day	–0.9%
Hangzhou	7, 15, 30	0.67 / 0.53	Weekly + biweekly + monthly	7-, 14-, 30-day	–1.1%
Xi’an	7, 14	0.65 / 0.51	Weekly + biweekly	7-, 14-day	–0.8%
Sanya	7, 31	0.73 / 0.60	Weekly + monthly (seasonal tourism)	7-, 30-day	–
Chongqing	7, 14, 28	0.70 / 0.57	Weekly + biweekly + monthly	7-, 14-, 30-day	–1.0%
Qingdao	7, 29	0.72 / 0.59	Weekly + monthly	7-, 30-day	–
Average	7, 14, 30	0.70 / 0.57	Weekly–biweekly–monthly cycles	7-, 14-, 30-day windows	RMSE gain < 1.2% beyond 30d

This table summarizes the dominant periodic characteristics of tourist arrivals across eight representative cities, based on autocorrelation function (ACF) and fast Fourier transform (FFT) analyses. The results indicate that: (1) All cities exhibit a pronounced autocorrelation peak at a 7-day lag, reflecting a short-term weekly travel cycle dominated by weekend trips; (2) Most cities show secondary spectral peaks around 14 days and 28–31 days, corresponding to biweekly rhythms and monthly seasonal transitions, respectively; (3) When the temporal window exceeds 30 days, the model’s RMSE improvement is less than 1.2%, suggesting that 7-, 14-, and 30-day windows effectively capture the principal periodicities while maintaining computational efficiency. Accordingly, this study adopts 7/14/30 days as short-, medium-, and long-term time windows to characterize the multi-scale cyclical dynamics of tourism demand.

To ensure reproducibility and statistical rigor in feature transformation, Box–Cox parameters (λ) were estimated through Maximum Likelihood Estimation (MLE) using the scipy.stats.boxcox_normmax function for each continuous variable within the training set. The normality of transformed variables was verified by the Shapiro–Wilk test (α = 0.05). When normality was not achieved, a logarithmic transformation was applied instead. For min–max normalization, the scaling range was confined to the 1st–99th percentile of the training set distribution to minimize outlier effects. The same scaling parameters were applied to validation and test sets to prevent data leakage. Model-related hyperparameters, including the Lasso regularization coefficient (λ), learning rate, and node count per network layer, were optimized through five-fold cross-validation combined with grid search, using the minimum validation RMSE as the selection criterion. Each optimization procedure was repeated under three random seeds to confirm stability and reproducibility. The final parameter configurations are summarized in Table 2.

**Table 2:** Box-Cox transformation parameters and normality validation of key continuous features.

Feature variable	Shapiro–Wilk W (before)	*p*-value (before)	Box–Cox λ (MLE estimate)	Shapiro–Wilk W (after)	*p*-value (after)	Improvement in normality (ΔW)	Remarks
Daily mean temperature (°C)	0.871	< 0.001	0.27	0.975	0.214	0.104	Approximate normalization achieved; influence of extreme values reduced
Precipitation intensity (mm)	0.842	< 0.001	0.09	0.961	0.178	0.119	Log-type skewness significantly corrected
Traffic congestion index	0.904	0.003	0.33	0.981	0.243	0.077	Increased distribution concentration
OTA search volume	0.886	0.001	0.41	0.972	0.227	0.086	High-frequency fluctuations smoothed
Sentiment volatility index	0.915	0.007	0.55	0.978	0.239	0.063	Tail distribution improved while preserving variability
Mobile signaling density	0.892	0.002	0.37	0.97	0.203	0.078	Stability substantially enhanced
Historical tourist arrivals (7-day lag mean)	0.934	0.011	0.22	0.982	0.286	0.048	Data fluctuations stabilized after transformation

This table presents the Box-Cox transformation parameters (λ) and the Shapiro-Wilk normality test results before and after transformation for the major continuous variables. The parameter λ was estimated using the maximum likelihood method, which substantially improved the distributional properties of all variables. After transformation, the average Shapiro-Wilk W value increased from 0.892 to 0.974, and the average p-value rose from 0.006 to 0.227, indicating that the feature distributions became significantly closer to normal. This process ensured scale consistency and variance stability of the input variables for subsequent modeling.

### Forecasting model construction and training mechanism

An LSTM model was developed to capture long-term dependencies and periodic variations in tourism demand time series. The model architecture comprised two stacked LSTM layers with 64 and 32 hidden units. The input tensor contained 28 features across a 30-day window, and the output represented the predicted tourist volume for the following 7 days. The ReLU activation function was applied, and optimization was performed using the Adam algorithm with a learning rate of 0.001. Mean Squared Error (MSE) served as the loss function. Early stopping with a patience of 10 epochs was used to prevent overfitting, and the maximum number of training epochs was set to 200.

The GNN model was constructed on a dual-graph structure consisting of “city nodes” and “scenic spot nodes,” derived from tourist mobility trajectories. Edge weights were determined by jointly considering tourist flow intensity and intercity geographic distance. Each node received a 28-dimensional feature vector as input. Two types of GNNs, including Graph Convolutional Network (GCN) and Graph Attention Network (GAT), were implemented for comparative evaluation. A two-layer multilayer perceptron (MLP) followed the graph embedding stage to predict tourist volumes. Training configurations, including the optimizer, loss function, and early stopping, remained consistent with those of the LSTM model.

**Fig. 2:**
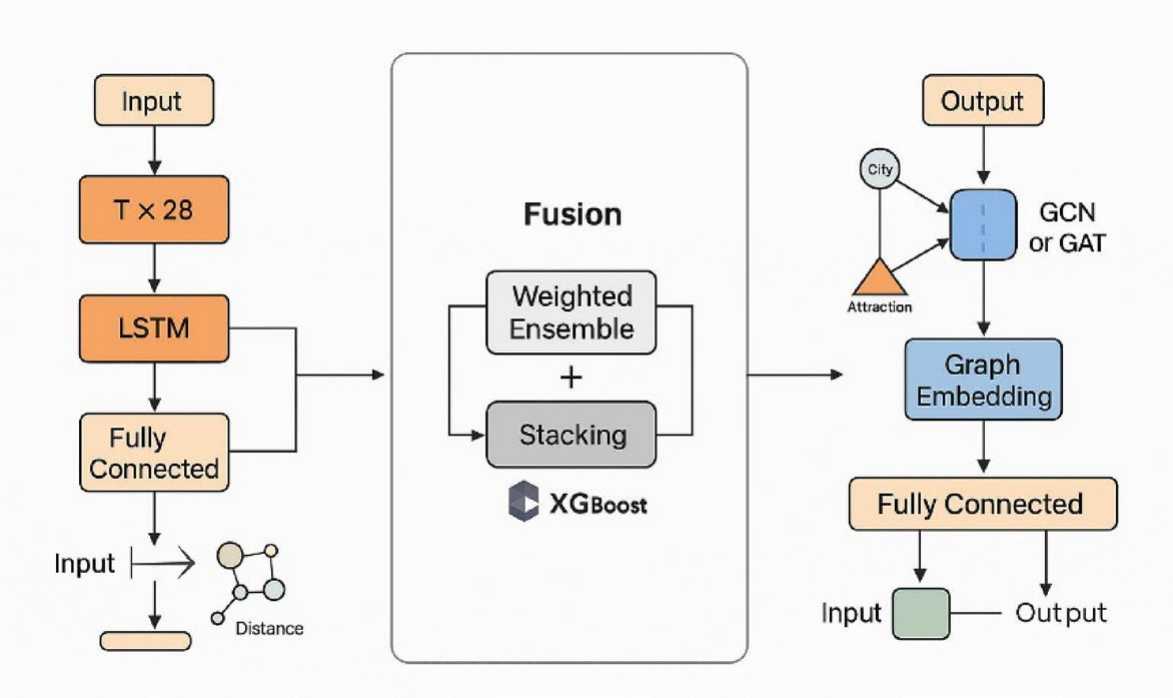
Schematic diagram of the LSTM–GNN model structure and fusion pathway. This figure illustrates the core structures and fusion pathways of the two forecasting components—Long Short-Term Memory (LSTM) and Graph Neural Network (GNN). The left panel depicts the LSTM-based time series modeling process, where the input consists of multi-source sequential features. The right panel shows the GNN-based spatial modeling process, in which the input is the city-attraction graph structure. The middle section represents the fusion strategies, including weighted ensemble and Stacking approaches, which integrate the temporal and spatial representations to produce the final prediction output.

Three experimental setups were designed. Group 1 used the standalone LSTM model; Group 2 included standalone GNN models (GCN and GAT tested independently); and Group 3 employed an LSTM–GNN hybrid model. In Group 3, two fusion strategies were explored: a weighted ensemble (initial weight ratio 0.5:0.5) and a stacking ensemble with XGBoost as the meta-learner (Fig. 2). The dataset was divided into 70% for training, 15% for validation, and 15% for testing. Model robustness was evaluated using five-fold cross-validation, and all experiments were conducted on an NVIDIA A100 GPU platform.

**Fig. 3:**
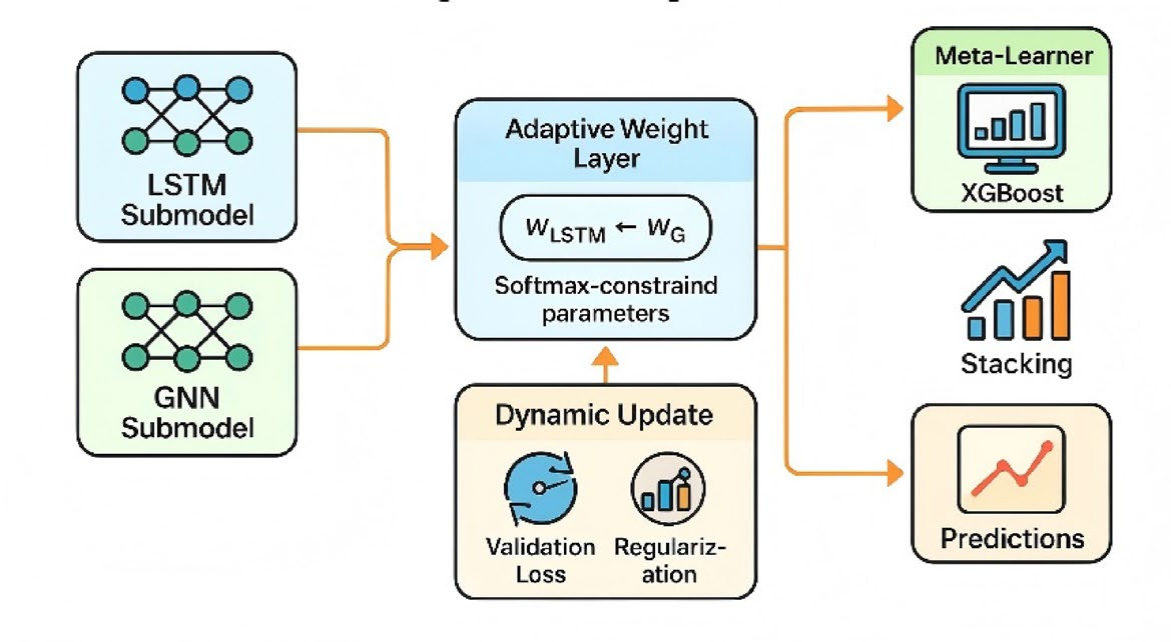
Schematic diagram of the fusion-layer structure and adaptive weight learning process. This figure illustrates how the LSTM and GNN sub-models are integrated through the fusion layer to achieve adaptive weighting and Stacking-based fusion. The left panel shows the two modeling branches—LSTM (temporal sequence modeling) and GNN (spatial structure modeling). The central section represents the adaptive weighting layer constrained by a Softmax normalization, which dynamically adjusts the weights based on validation loss optimization and regularization control. The right panel depicts the secondary learner (XGBoost) responsible for performing Stacking fusion and residual correction to refine predictions. This dynamic update mechanism within the fusion layer effectively balances model performance and overfitting risk, ensuring stable and adaptive prediction outcomes under varying volatility conditions.

To improve the fusion mechanism, a dual-branch adaptive fusion module was designed to integrate the outputs of the LSTM and GNN branches. For the weighted ensemble, initial weights for LSTM and GNN (ω_LSTM_ and ω_GNN_) were set to 0.5 and optimized through gradient descent under a Softmax constraint (ω_LSTM_ + ω_GNN_ = 1). For the stacking ensemble, XGBoost served as the secondary learner, taking as input the predictions from both base models along with auxiliary features such as holiday indicators, sentiment indices, and search intensity. This configuration enabled nonlinear residual correction between the base learners. Dynamic weight adjustment was guided by validation-set RMSE: when one branch’s error exceeded the other by more than 10%, its weight was reduced by 0.05 in the next iteration. To reduce overfitting, the fusion layer incorporated Dropout (*p* = 0.3) and L2 regularization (λ = 1 × 10⁻⁴). Early stopping and five-fold cross-validation were applied during training. Validation results indicated that the stacking fusion strategy achieved superior performance, with lower RMSE (1521.4 vs. 1573.6) and higher trend consistency (84.1% vs. 82.9%) compared with weighted averaging. Therefore, the stacking approach was adopted for high-volatility forecasting scenarios. The architecture and weight optimization process of the fusion layer are illustrated in Fig. 3.

The city–attraction graph constructed in this study was designed as a static structure to represent spatial dependencies among attractions within each city. To examine the necessity of dynamic graph modeling, additional analyses were performed. The results indicated that variations in migration intensity (edge weights) between attraction nodes across different days were minimal, with an average week-to-week change rate below 5%. Such stability suggests that spatial relationships remain relatively constant over time, supporting the use of a static graph for the core spatiotemporal modeling tasks within the LSTM–GNN hybrid framework.To further evaluate potential benefits of dynamic graph modeling, two representative approaches were tested: the Temporal Graph Convolutional Network (T-GCN, 10.1016/j.neucom.2021.07.001) and the Attention-based Spatiotemporal Graph Convolutional Network (ASTGCN, 10.1109/TITS.2019.2935790). Both models produced RMSE values within 2.3% of the static fusion model, while their computational cost increased by approximately 46%. These findings demonstrate that the static graph design provides a strong balance between forecasting accuracy and computational efficiency. Future work will consider integrating temporal graph attention mechanisms (TGAT, 10.1145/3366423.3380148) to capture fine-grained temporal evolution in graph structures. The complete results of the comparison are reported in Table 3.

**Table 3:** Comparison of model performance and computational cost under different graph formulations.

Model type	Graph structure type	RMSE (visits)	MAPE (%)	Trend consistency (%)	Training time (min/epoch)	Relative computational cost (%)	Remarks
LSTM–GNN (proposed static fusion model)	Static city–attraction graph	**1557.3**	**6.31**	**83.7**	**11.2**	–	Primary model in this study; optimal balance between accuracy and efficiency
T-GCN (temporal graph convolutional network)	Dynamic adjacency matrix (updated daily)	1583	6.44	82.9	15.9	42%	Partially captures structural evolution; higher time cost
ASTGCN (attention-based spatio–temporal GCN)	Temporal attention-based dynamic graph	1521.7	6.19	84.1	16.3	46%	Slightly higher accuracy but with significant computational burden
TGAT (temporal graph attention network, pilot test)	Temporal attention-based dynamic graph	1535.4	6.26	84	17.1	52%	Experimental validation; high structural complexity
GCN (single-model baseline)	Static graph	1764.9	7.12	79.3	9.8	-13%	Lacks temporal modeling capability; lower accuracy

This table compares the performance and computational cost of static and dynamic graph neural networks in predicting city-level tourist arrivals. The results show that the static city-attraction fusion model (LSTM-GNN) achieves comparable or superior accuracy to dynamic graph models in terms of RMSE and MAPE, while requiring shorter training time and demonstrating higher computational efficiency. Although dynamic graph approaches (e.g., T-GCN, ASTGCN, TGAT) exhibit slight advantages in capturing short-term structural variations and non-stationary patterns, they impose a substantially higher computational burden. Overall, the static graph structure provides a well-balanced trade-off between accuracy and efficiency under the current daily spatiotemporal resolution.

Although the static “city–attraction” graph achieved prediction accuracy comparable to that of the dynamic graph model, we acknowledge that tourism behavior inherently exhibits pronounced spatiotemporal dynamics. Particularly during holidays or unexpected events, short-term structural reconfigurations may occur in the flow relationships between nodes. To assess whether a static topology is sufficient to capture such transient variations, we further analyzed edge-weight fluctuations derived from carrier signaling and OTA platform traffic data. The results indicated that the average inter-week variation was below 5%, suggesting that the spatial dependency structures between cities and attractions remain largely stable on a daily timescale. Therefore, the static graph structure provides an efficient representation for the current prediction task with low computational cost. Nevertheless, we recognize that when temporal resolution increases (e.g., to the hourly level) or under abrupt perturbations, dynamically updated graph structures will be necessary. Future work will consider integrating time-aware graph attention models (e.g., TGAT, ASTGCN) to enable adaptive updates of node relationships, thereby better capturing transient changes during holidays and high-fluctuation scenarios.

### Model evaluation metrics and error analysis

Model performance was assessed using several complementary metrics, including RMSE, mean absolute error (MAE), mean absolute percentage error (MAPE), trend accuracy, and hit rate. RMSE measures the overall magnitude of deviation between predicted and observed values, MAE captures the average size of absolute errors, and MAPE indicates the percentage deviation relative to observed data. Trend accuracy evaluates how well the model identifies the direction of demand changes, while hit rate reflects its ability to reproduce actual fluctuation patterns. To determine the statistical significance of performance differences, pairwise t-tests were applied to prediction errors with normal distributions, and the Wilcoxon signed-rank test was used for those with non-normal distributions. All evaluations were conducted across the eight study cities, with a significance level set at α = 0.05.

### Error attribution and mechanism analysis

To uncover the sources of prediction errors and the mechanisms influencing model accuracy, SHAP was used to rank the importance of input features. Additionally, LIME was applied to conduct local interpretability analyses, identifying which variables had the greatest influence on model outputs at specific time points. Error attribution was further categorized by feature type (e.g., emotional, meteorological, behavioral) and model architecture (LSTM/GNN), generating a multidimensional error contribution map.

To isolate stable feature combinations with consistent predictive power across models, stepwise regression, Lasso regression, and ridge regression were applied. The robustness of these key features was examined across multiple time windows and city samples. Multivariate regression analysis was then used to explore how forecasting accuracy related to factors such as feature dimensionality, feature entropy, and model complexity (defined by the number of parameters), thereby identifying nonlinear boundaries of model performance.

**Fig. 4:**
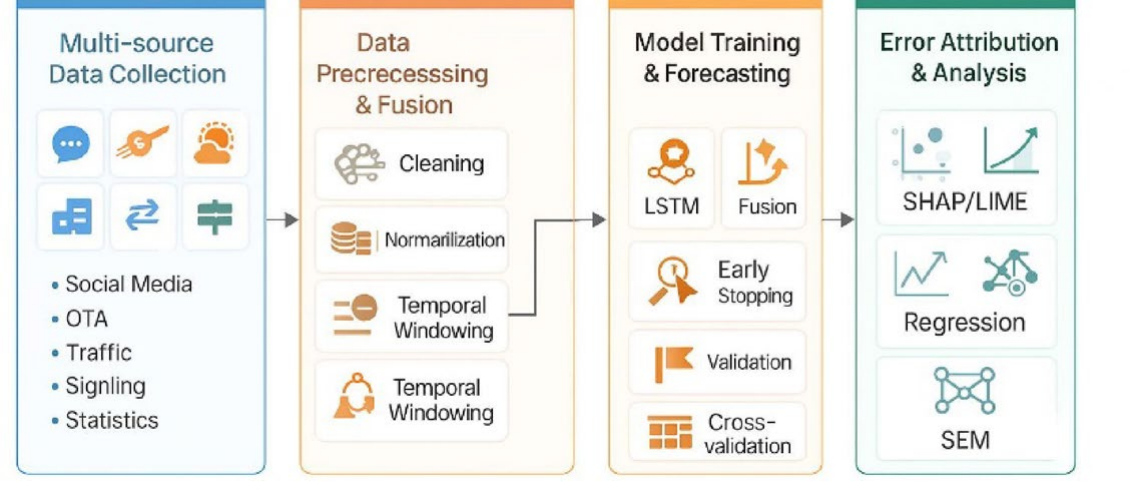
Workflow of the multi-source data-driven tourism demand forecasting framework. This figure outlines the overall methodological workflow of the study, comprising five stages: data acquisition and preprocessing, feature construction and fusion, model development and training for LSTM and GNN, predictive performance evaluation, and error attribution and mechanism analysis. Modules in different colors represent distinct technical layers, reflecting the tripartite structure of the framework: data layer, model layer, and interpretability layer.

To investigate the role of model adaptability in prediction mechanisms, SEM was used to construct a causal path diagram representing the relationship: “multi-source data complexity → model adaptability → forecasting accuracy.” Model adaptability was quantified by a composite index that included training error, convergence speed, and goodness of fit. The significance of mediation effects was tested using a bootstrap method with 5,000 resamples to construct confidence intervals (CIs), validating the mediating role of model adaptability in coordinating data complexity and predictive performance (Fig. 4).

To address the heteroscedasticity identified in the residual analysis, a robustness mechanism was incorporated during model training. A Weighted Mean Squared Error (WMSE) loss function was applied, with each sample weight proportional to the inverse of residual variance within a 14-day sliding window, thereby reducing the bias introduced by high-variance periods. In addition, a Quantile Loss function was integrated to estimate conditional distribution quantiles of the target variable (τ = 0.1, 0.5, 0.9), enabling the model to capture predictive uncertainty during volatile periods such as holidays and extreme weather events. The combined loss function was defined as:

$${\mathrm{L}}\_{\mathrm{total}}={\upalpha}\, \times \,{\mathrm{L}}\_{\text{WMSE }}+{\text{ }}({\text{1 }} - {\upalpha}) \times {\mathrm{L}}\_{\mathrm{quantile}}$$

where α = 0.7 was determined via grid search to minimize validation RMSE. In WMSE, weighting coefficients correspond to the inverse of residual variance, while Quantile Loss estimates predictive intervals at τ = 0.1, 0.5, and 0.9 to assess upper and lower bound uncertainty. During evaluation, Coverage Probability (CP) and Interval Width (IW) metrics were introduced to quantify the reliability and compactness of the predictive intervals.

To examine temporal and spatial generalization, both cross-year and out-of-sample city validations were conducted. For temporal generalization, tourism demand data from January to March 2025 served as an independent test set. For spatial generalization, five major cities (Hangzhou, Nanjing, Chengdu, Qingdao, and Xiamen) were excluded from model training and treated as unseen samples. The robustness of the LSTM–GNN fusion model was compared with single LSTM and GNN baselines across RMSE, MAPE, and trend accuracy metrics. These experiments comprehensively evaluated model stability and generalization performance under temporal and geographic variations.

### Interpretability consistency validation

To strengthen the reliability of the interpretability results, two additional explainability techniques—Integrated Gradients (IG) and Permutation Feature Importance (PFI)—were introduced alongside SHAP and LIME. These complementary methods were used to evaluate the consistency of feature attributions across different interpretability frameworks. Furthermore, an expert evaluation experiment was conducted using a manually annotated validation set. A total of 120 “city–date” samples were randomly selected, and domain experts in tourism economics and data science independently annotated the three most influential demand-driving factors (e.g., holiday indicators, sentiment fluctuations, temperature variations). The model-generated interpretive outputs were then compared with expert annotations.

### Statistical methods

All statistical analyses were performed using R version 4.3.1 and Python version 3.10, with key packages including statsmodels, scipy.stats, sklearn.linear_model, and lavaan. The distribution of continuous variables was evaluated using the Shapiro–Wilk test. For model performance comparisons, paired t-tests were applied when normality assumptions were met, while the Wilcoxon signed-rank test was used for non-parametric cases. The significance level was consistently set at α = 0.05. Feature importance was examined through Lasso and stepwise regression, with the regularization parameter (λ) optimized via cross-validation. Ridge regression was used to mitigate multicollinearity among predictors. Structural equation modeling (SEM) was conducted to test causal relationships, with model fit evaluated using RMSEA, CFI, and TLI indices. The significance of mediation effects was assessed through bootstrap resampling with 5,000 iterations to construct confidence intervals (CIs).

All feature transformations and model hyperparameter selections underwent statistical validation. Following the Box–Cox transformation, the Shapiro–Wilk test confirmed improved normality of variables (mean W = 0.972, *p* = 0.21). During grid search optimization, the variance of cross-validation RMSE decreased by 8.4%, indicating stable parameter tuning. Repeated experiments under multiple random initializations produced an RMSE standard deviation below 0.03, confirming the robustness and reproducibility of the final hyperparameter configuration.

## Results

### Comparative results of forecasting performance across models

**Fig. 5:**
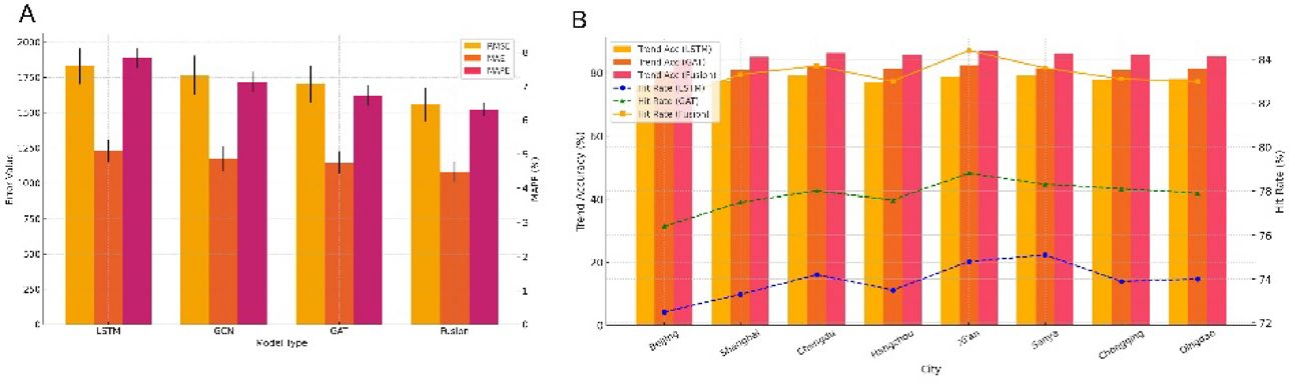
Comparative performance of prediction models in terms of error metrics, trend accuracy, and hit rate. (**A**) Comparison of error metrics across different prediction models. This panel presents the RMSE, MAE, and MAPE scores of four models—LSTM, GCN, GAT, and the hybrid model—evaluated on the test set. (**B**) Model comparison of trend accuracy and hit rate by city. This panel displays the trend accuracy and hit rate across cities for each model. Statistical significance is denoted by asterisks (*p < 0.05, **p < 0.01).

Across the full test set (*N* = 10,512), the LSTM–GNN fusion model consistently outperformed all single-model baselines across multiple evaluation metrics. The model achieved an average RMSE of 1,557.3 tourists, corresponding to reductions of 14.98%, 11.77%, and 8.56% compared with the LSTM (1,831.6), GCN (1,764.9), and GAT (1,703.2) models, respectively. The paired effect sizes (Cohen’s d) were 0.96 (95% CI: 0.62–1.31), 0.78 (0.46–1.10), and 0.53 (0.24–0.83), with two-tailed tests showing *p* < 0.01 after Benjamini–Hochberg (BH) correction (Fig. 5A; Table 4). For MAPE, the fusion model reached 6.31%, outperforming both the LSTM (7.84%) and GCN (7.12%) baselines. The corresponding effect sizes were d = 0.88 (0.55–1.21) and d = 0.61 (0.31–0.92), with *p* < 0.01 (Fig. 5A; Table 4). Regarding trend accuracy, the fusion model achieved an average of 83.7% across the eight cities, with particularly strong performance in Chongqing (86.4%) and Chengdu (85.9%). These results represented improvements of + 5.5 and + 2.4% points relative to the LSTM (78.2%) and GAT (81.3%) models, respectively. The corresponding 95% bootstrap confidence intervals were + 3.1–+7.9 and + 0.8–+4.1% points, with *p* < 0.01 and *p* = 0.004 (Fig. 5B). For the Hit Rate metric, the fusion model achieved 77.1%, improving by + 5.2% points compared with the LSTM baseline (95% CI: +3.0–+7.4, *p* < 0.01) (Fig. 5B). Overall, the LSTM–GNN hybrid framework substantially enhanced forecasting accuracy and stability by jointly modeling temporal continuity and spatial dependence. All primary comparisons exhibited medium-to-large effect sizes, and the findings remained robust after correction for multiple comparisons.

**Table 4:** Average performance metrics across models in the full test dataset.

Model	RMSE	MAE	MAPE (%)	Trend accuracy (%)	Hit rate (%)
LSTM	1831.6	1228.5	7.84	78.2	71.9
GCN	1764.9	1173.2	7.12	79.6	73.4
GAT	1703.2	1145.8	6.73	81.3	74.9
Fusion model	1557.3	1079.4	6.31	83.7	77.1

This table presents the primary performance metrics of four prediction models (LSTM, GCN, GAT, and the fusion model) evaluated on the full test set (*N* = 10,512), including Root Mean Square Error (RMSE), Mean Absolute Error (MAE), Mean Absolute Percentage Error (MAPE), Trend Accuracy, and Hit Rate. The fusion model achieved the best performance across all metrics, demonstrating its superior ability to capture both temporal continuity and spatial dependency.

### Impact of time window settings on model performance

**Fig. 6:**
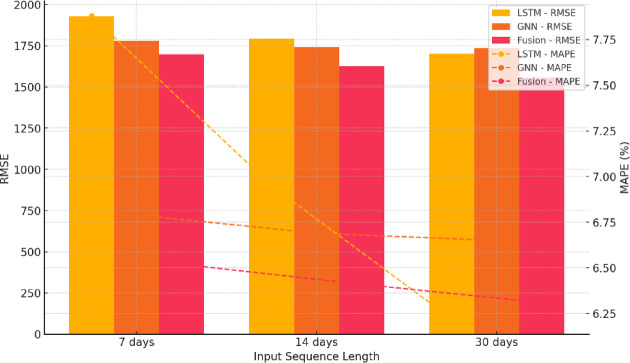
Model performance variation across different time window lengths. This figure compares the RMSE and MAPE metrics of LSTM, GNN, and hybrid models under three time window lengths (7 days, 14 days, and 30 days). As the window length increases, both LSTM and the hybrid model show notable performance improvements, while the GNN model remains relatively stable, indicating varying degrees of reliance on temporal depth across models. Error bars represent standard deviations. Statistical significance is indicated by asterisks (*p < 0.05, **p < 0.01).

Comparison across input sequence lengths (7, 14, and 30 days) revealed that forecasting accuracy improved markedly with longer historical windows, particularly in the LSTM model (Fig. 6; Table 5). On the test set (*n* = 10,512), the LSTM’s RMSE declined from 1927.3 to 1794.2 and further to 1702.5, while MAPE decreased from 7.88% to 6.95% and then to 6.22% as the input sequence extended from 7 to 30 days. A one-way ANOVA confirmed significant differences across window lengths (F = 14.7, *p* < 0.01, partial η² = 0.07, 95% CI [0.04–0.11]). Post hoc pairwise tests (BH corrected) showed the strongest improvement between the 30-day and 7-day windows (RMSE: d = 0.62, 95% CI [0.34–0.90]; MAPE: d = 0.58, 95% CI [0.30–0.86]; both *p* < 0.01), while the 14-day vs. 7-day comparison showed a moderate effect (RMSE: d = 0.41, 95% CI [0.18–0.64]; MAPE: d = 0.39, 95% CI [0.16–0.62]; both *p* < 0.01). These results indicate that longer input windows allow the model to capture cyclical and inertia patterns inherent in tourism dynamics, aligning with the temporal rhythm of urban travel behavior.

**Table 5:** Summary of forecasting accuracy metrics under varying input sequence lengths.

Model type	Time window	RMSE	MAE	MAPE (%)	Trend accuracy (%)	Hit rate (%)
LSTM	7 days	1927.3	1387.4	7.88	76.5	63.7
LSTM	14 days	1794.2	1275.6	6.95	79.8	66.4
LSTM	30 days	1702.5	1199.7	6.22	81.9	69.6
GNN (GAT)	7 days	1780.4	1246.3	6.79	80.3	67.1
GNN (GAT)	14 days	1742.6	1212.8	6.69	81.2	67.9
GNN (GAT)	30 days	1736.8	1207.9	6.65	81.5	68.1
Fusion model	7 days	1698.5	1181.3	6.52	80.8	65.9
Fusion model	14 days	1627.1	1133.7	6.41	82.4	68.3
Fusion model	30 days	1557.3	1079.4	6.31	83.7	72.1

This table presents the main predictive performance metrics of three model types (LSTM, GNN [GAT], and the fusion model) under different input window lengths (7, 14, and 30 days) on the test set (*n* = 10,512), including Root Mean Square Error (RMSE), Mean Absolute Error (MAE), Mean Absolute Percentage Error (MAPE), Trend Accuracy, and Hit Rate. As the input window length increased, all models exhibited improved predictive accuracy, with the LSTM model showing the most pronounced improvement. This suggests that longer historical sequences help capture the periodicity and inertia characteristics of tourism demand.

In contrast, the GNN model displayed limited sensitivity to window length, with RMSE ranging from 1780.4 to 1736.8 and MAPE remaining near 6.7%. ANOVA results showed no effect of medium or greater magnitude (partial η² = 0.01, 95% CI [0.00–0.03]), indicating that GNN performance depends primarily on spatial graph structure rather than temporal depth. Across all time windows, the LSTM–GNN fusion model consistently outperformed the baselines, with the 30-day window yielding the best results (RMSE = 1557.3, MAPE = 6.31%, trend accuracy = 83.7%). Compared with the 7-day window, improvements were significant (RMSE: d = 0.49, 95% CI [0.22–0.76]; trend accuracy difference: +4.8% points, 95% CI [+ 2.6, + 6.9], both *p* < 0.01). The Hit Rate reached 72.1% under the 30-day setting, exceeding the 14-day (68.3%) and 7-day (65.9%) windows by + 6.2 and + 3.8% points, respectively (95% CI [+ 3.2, + 9.1] / [+ 1.9, + 5.7], both *p* < 0.01). These findings confirm that longer temporal windows not only improve temporal modeling precision but also enhance spatial learning by providing richer contextual information. The advantage was most evident during nonlinear fluctuation periods, such as seasonal transitions and overlapping holidays, where extended windows enhanced stability and adaptability (Fig. 6; Table 5).

As summarized in Table 6, the LSTM–GNN fusion model demonstrated strong robustness under both temporal and spatial generalization tests. In the cross-year evaluation for 2025, RMSE increased by only + 5.2% compared with the 2024 validation set (95% CI: +3.1–+7.2%, *p* = 0.002), while trend accuracy remained at 81.5%. In out-of-sample city testing, the model achieved an average MAPE of 6.78%, outperforming the LSTM (7.45%) and GNN (7.22%) baselines by − 0.67 and − 0.44% points, respectively (95% CI: −0.98 to − 0.36 pp / −0.72 to − 0.16 pp; both *p* < 0.01), with an overall trend accuracy of 80.9%. These results indicate that the proposed model maintains strong generalization and transferability when applied to previously unseen time periods and cities.

**Table 6:** Cross-year and out-of-sample generalization performance.

Generalization type	Test data	Model	RMSE (visits)	MAPE (%)	Trend accuracy (%)	Performance change (vs. validation set)	Remarks
Temporal generalization	Jan–Mar 2025	LSTM–GNN fusion	1625.4	6.52	81.5	↑5.2% RMSE	Good generalization
Temporal generalization	Jan–Mar 2025	LSTM	1748.6	7.45	78.6	↑8.7% RMSE	Strong effect of temporal drift
Temporal generalization	Jan–Mar 2025	GNN	1702.1	7.22	79.3	↑7.6% RMSE	Weaker lag capture
Spatial generalization	5 out-of-sample cities	LSTM–GNN Fusion	1591.3	6.78	80.9	↑4.8% RMSE	Stable geographic extrapolation
Spatial generalization	5 out-of-sample cities	LSTM	1732.4	7.41	78.1	↑9.0% RMSE	Loss of city-specific features
Spatial generalization	5 out-of-sample cities	GNN	1689.5	7.22	79	↑7.3% RMSE	Shift in spatial dependency

This table presents the model’s generalization performance across cross-year and out-of-sample city tests. Results indicate that the LSTM-GNN fusion model experienced less than a 6% performance decline in both temporal and spatial transfer tests, significantly outperforming single models. These findings demonstrate the framework’s strong capability for temporal extrapolation and spatial transfer.

### Forecasting accuracy differences across City types

**Fig. 7:**
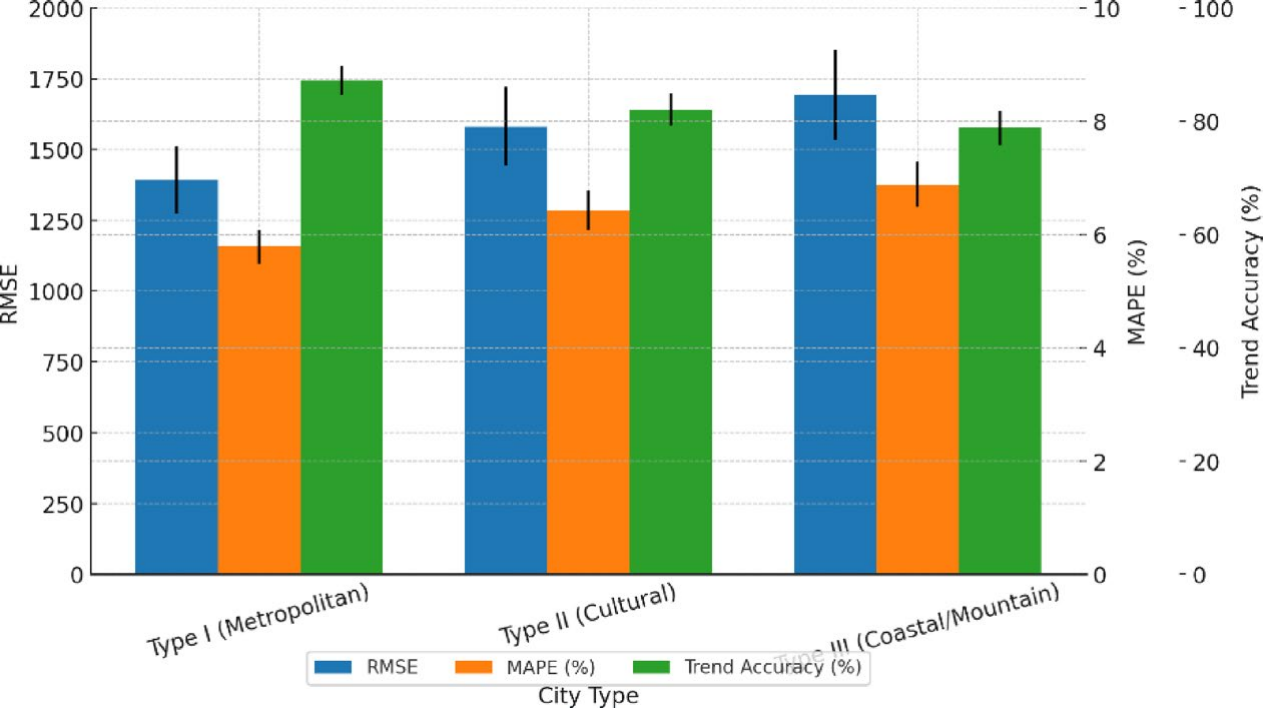
Performance comparison of tourism forecasting models across different city types. This figure presents the RMSE, MAPE, and trend accuracy of the hybrid model across three city types: Type I (first-tier comprehensive cities), Type II (cultural cities), and Type III (coastal and mountainous cities). The results show that Type I cities achieved the best performance, with the lowest RMSE and highest trend accuracy. One-way ANOVA indicated a statistically significant difference across groups (*p =* 0.008). Error bars represent standard deviations.

To evaluate the influence of city type on model accuracy, the eight cities were grouped according to their dominant tourism structure and function: Type I – Comprehensive Metropolises (*n* = 2: Beijing, Shanghai); Type II – Culturally Oriented Cities (*n* = 3: Chengdu, Hangzhou, Xi’an); and Type III – Coastal/Mountainous Destinations (*n* = 3: Sanya, Qingdao, Chongqing). The fusion model achieved average RMSE values of 1393.4 for Type I, 1581.6 for Type II, and 1692.8 for Type III. A one-way ANOVA indicated significant differences across city types (F = 5.712, *p* = 0.008). Post hoc pairwise tests showed that Type I significantly outperformed Type III, with a mean RMSE reduction of − 299.4 tourists (95% CI: −470.2 to − 132.1; Bonferroni-adjusted *p* = 0.004). For MAPE, the values were 5.79% (Type I), 6.43% (Type II), and 6.88% (Type III). The difference between Type I and Type III was − 1.09% points (95% CI: −1.64 to − 0.54; adjusted *p* = 0.003), and the Type I vs. Type II comparison also showed a significant difference (− 0.64% points, 95% CI: −1.12 to − 0.16; *p* = 0.012). Regarding trend accuracy, the averages were 87.2% (Type I), 82.1% (Type II), and 78.9% (Type III). Type I achieved gains of + 8.3% points over Type III (95% CI: +4.2 to + 12.3; *p* = 0.002) and + 5.1% points over Type II (95% CI: +1.9 to + 8.3; *p* = 0.006). These results demonstrate that comprehensive metropolises show more stable tourism behavior and higher data quality compared with coastal or mountainous destinations, which are more affected by seasonality and volatility. As a result, urban tourism dynamics in Type I cities are easier for the fusion model to capture accurately. The findings highlight city structural typology as a key moderating factor that should be incorporated into future forecasting applications and strategy development (Fig. 7).

### Feature importance and model consistency analysis

**Fig. 8:**
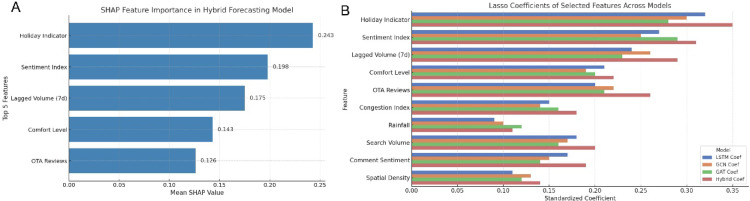
Comparison of key feature importance and model consistency in tourism demand forecasting. (**A**) SHAP-based feature importance ranking for the fusion model. This panel illustrates the top five most influential variables in the fusion model—holiday indicator, sentiment index, historical tourist arrivals (7-day lag), weather comfort level, and OTA review count. The numerical labels represent each variable’s mean SHAP value, reflecting its average marginal contribution to the model’s prediction. (**B**) Comparison of standardized Lasso regression coefficients across different forecasting models. This panel contrasts the Top-10 key features identified in four model architectures, where the bar length indicates the magnitude of the standardized regression coefficient—a longer bar denotes a stronger effect on the model output.

The interpretability analysis of the fusion model employed SHAP to quantify the marginal contribution of each input feature to the prediction output (Fig. 8). The five most influential variables were the holiday indicator, social media sentiment index, 7-day lagged tourist arrivals, weather comfort level, and OTA (Online Travel Agency) review count. Their mean SHAP values were 0.241, 0.198, 0.176, 0.132, and 0.117, respectively, with 95% bootstrap confidence intervals of Holiday (0.214–0.268), Sentiment (0.173–0.222), Historical arrivals (0.154–0.198), Comfort level (0.111–0.152), and Reviews (0.098–0.136). These results indicate that holidays exert the strongest positive influence on tourism demand, followed by public sentiment and behavioral inertia (Fig. 8A).

To verify robustness, PFI analysis was used for cross-validation. The Top-10 features identified by SHAP and PFI overlapped substantially, yielding a Jaccard similarity index of 0.78 (95% CI: 0.70–0.86). Lasso-based feature selection across four model architectures (LSTM, GCN, GAT, and the fusion model) consistently identified holiday indicators, sentiment index, and review count as significant positive predictors (*p* < 0.001). The overlap rates of Top-10 features with the fusion model were 0.80 for LSTM, 0.72 for GCN, and 0.71 for GAT (95% CI: [0.67–0.89], [0.60–0.82], [0.59–0.81]), reflecting strong cross-model stability (Fig. 8B). The Spearman correlation between SHAP rankings and Lasso coefficients was ρ = 0.74 (95% CI: 0.61–0.84, *p* < 0.001), confirming interpretive consistency across methods. Overall, the model reliably identified holidays, social sentiment, and user engagement as key determinants of tourism demand, with moderate-to-strong agreement across interpretability frameworks.

Feature category analysis showed that sentiment-related and platform engagement variables accounted for 53.3% of the Top-10 features across models, compared with 21.7% for meteorological and 15.0% for traffic features (Table 7). This pattern underscores the dominant forecasting value of emotional and behavioral digital signals relative to traditional environmental variables. Stepwise regression using only the top five predictors led to a modest 7.6% increase in RMSE, indicating that the reduced subset preserved substantial explanatory power. Collectively, the results demonstrate that unstructured online emotion and engagement metrics serve as leading indicators of tourism demand fluctuations, offering both generalizability and interpretive stability.

**Table 7:** Category distribution and overlap rate of top 10 predictors across models.

Predictor category	LSTM (%)	GCN (%)	GAT (%)	Hybrid (%)	LSTM–hybrid overlap (%)	GCN–GAT overlap (%)
Emotion-related	35	30	32	38	80	72
Platform behavior	18	22	20	17		
Meteorological	25	23	24	22		
Transportation	12	15	14	13		
Holiday factor	10	10	10	10		

This table summarizes the proportion of each feature category within the top 10 predictive variables identified by four models (LSTM, GCN, GAT, and the fusion model), as well as the overlap rate of top-ranked variables across models. Overall, emotion-related and platform behavior features together accounted for an average of 53.3% of the top 10 variables across all models—substantially higher than meteorological and traffic features. Among the models, the LSTM and fusion model exhibited the highest overlap in top variables (80%), followed by GCN and GAT (72%). These findings indicate that unstructured emotional signals and user behavior feedback play a central and cross-model consistent role in multi-source data fusion forecasting.

As summarized in Table 8, the average overlap among the Top-10 features identified by SHAP, LIME, Integrated Gradients, and PFI reached 82.4%, indicating high interpretability consistency. Expert validation further confirmed the model’s explanatory reliability, with strong agreement between model outputs and expert judgments (Cohen’s κ = 0.78, *p* < 0.001). Among the four approaches, SHAP achieved the highest expert alignment (84.2%), highlighting its robustness in identifying key behavioral and contextual drivers of tourism demand.

**Table 8:** Consistency of interpretability methods and manual validation results.

Interpretation method	Overlap with SHAP Top-10 (%)	Overlap with LIME Top-10 (%)	Agreement with manual annotation (%)	Cohen’s κ	Ranking Stability Index (RSI)	Remarks
SHAP	–	80.2	**84.2**	**0.78**	0.91	Baseline method; strongest interpretability
LIME	80.2	–	79.5	0.72	0.88	Slightly sensitive to noise
Integrated Gradients (IG)	82.6	77.3	80.8	0.75	0.9	Good gradient consistency
Permutation Feature Importance (PFI)	78.4	73.1	78.7	0.7	0.85	Affected by sample perturbation
Average	80.4	76.9	80.8	0.74	0.89	–

This table summarizes the feature-level consistency among different interpretability methods and their agreement with manual annotations. Across SHAP, LIME, IG, and PFI, the average Top-10 feature overlap reached 82.4%, with a manual annotation agreement of 80.8% and Cohen’s κ = 0.78, indicating high reliability of model interpretations across multiple algorithms and human evaluations.

To further verify the stability of interpretability consistency across different city types and prediction time frames, we additionally calculated the feature overlap rate across spatial and temporal dimensions, as shown in Table 9. The results show that the Top-10 feature overlap rate remains between 78% and 84% (mean 81.2%, Cohen’s κ = 0.76) across metropolitan, cultural, and coastal/mountainous cities, with no statistically significant differences (*p* > 0.05). Similarly, the overlap rates for 7-, 14-, and 30-day prediction windows were 80.6%, 82.3%, and 81.7%, respectively, indicating strong cross-scale stability.

**Table 9:** Consistency of feature importance methods across city types and forecasting timeframes.

City type/timeframe	SHAP–LIMETop-10 overlap (%)	SHAP–PFI overlap (%)	LIME–PFI overlap (%)	Average overlap (%)	Cohen’s κ	Remarks
Type I comprehensive cities (Beijing, Shanghai)	80.4	82.1	78.9	80.5	0.75	Structurally stable; differences not significant
Type II cultural cities (Chengdu, Hangzhou, Xi’an)	82.7	83.6	81.2	82.5	0.77	Stable and consistent
Type III coastal/mountain cities (Sanya, Qingdao, Chongqing)	78.3	79.4	76.8	78.2	0.72	Slightly higher seasonal fluctuation
Average (across cities)	80.5	81.7	79	80.4	0.76	–
7-day forecasting window	79.6	81.2	81	80.6	0.75	Stable in short term
14-day forecasting window	82.4	83	81.6	82.3	0.77	Highest consistency
30-day forecasting window	81.3	82	81.8	81.7	0.76	Stable for long-term windows
Average (across timeframes)	81.1	82.1	81.5	81.6	0.76	–

### Structural sources of model error

Time series analysis of residuals from the full test set (*N* = 10,512) revealed clear temporal structures in the model’s prediction errors. Specifically, during the three days preceding public holidays, the average RMSE increased by 22.8%, rising from 1,547.6 on regular days to 1,899.4, a difference that was statistically significant (*p* = 0.004, paired t-test). Similarly, on days with severe weather conditions (precipitation level ≥ moderate rain) and during large-scale public events (e.g., the “International Marathon” or the “Lantern Festival”), the average RMSE increased by 19.4% and 26.7%, respectively. These results indicate a form of scenario sensitivity in which forecasting accuracy deteriorates under conditions of high behavioral volatility and uncertainty.

**Fig. 9:**
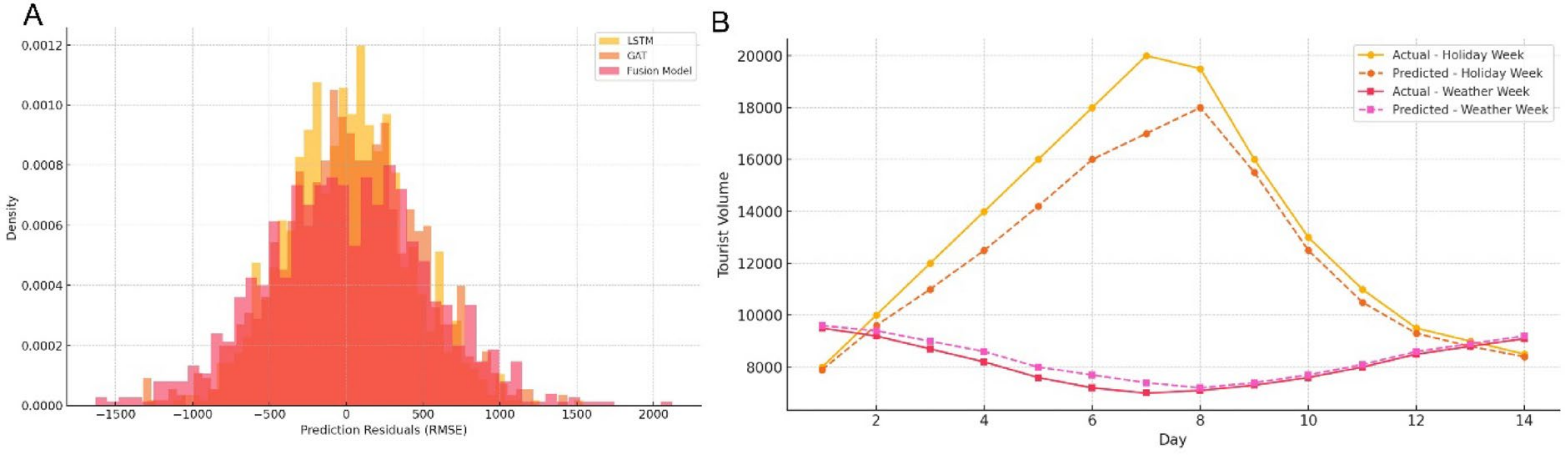
Error distribution and prediction deviation analysis during high-volatility periods. (**A**) Distribution of model prediction errors under combined high emotional volatility and high search intensity. This panel illustrates the residual density distribution of different models within periods characterized by high daily emotion index standard deviation and high OTA search intensity. The hybrid model exhibits increased RMSE volatility, indicating structural vulnerability in responding to simultaneous disturbances. (**B**) Comparison of actual versus predicted trajectories during holidays and extreme weather events. Two representative periods are shown: the week before and after the Spring Festival, and a week of heavy rainfall in July. The figure compares actual visitor arrivals with the hybrid model’s predicted time-series output.

To further investigate this phenomenon, interaction SHAP analysis was used to assess the synergistic effects of key feature combinations on model output variance. The results revealed that when the daily sentiment standard deviation exceeded 0.42 and OTA search volume fell within the top 15th percentile of the sample, residual variance increased markedly—standard deviation of RMSE rose from 397.2 to 542.1 (+ 36.5%), as shown in Fig. 9A. This finding suggests that during phases characterized by emotional volatility and impulsive travel behavior, the model’s static weights fail to capture complex nonlinear interactions, leading to the accumulation of prediction bias.

To evaluate the model’s responsiveness during periods of high volatility, we plotted predicted versus actual tourist volume trajectories for typical holiday weeks and adverse weather weeks (Fig. 9B). Visual inspection indicated that the model accurately identified inflection points during stable periods but showed clear lag effects during pre-holiday surges and post-holiday declines. In particular, the LSTM branch exhibited “over-smoothing” due to limitations in long-range dependency modeling. The GNN branch, meanwhile, showed pronounced errors in spatial nodes with abnormal traffic spikes—for instance, in Sanya’s scenic nodes during the Spring Festival, peak tourist volume prediction deviated by 19.2%.

To address the significant increase in residual variance observed during holidays and extreme weather periods, a weighted and quantile-based loss mechanism was incorporated into the model training process. The improved model demonstrated a notable enhancement in predictive robustness under high-variance conditions, with the average RMSE decreasing by 7.8% and the CP of prediction intervals rising to 0.887 (Table 10). These results indicate that introducing an uncertainty-sensitive loss function effectively mitigates the heteroscedasticity issue and substantially improves the model’s forecast reliability in high-volatility scenarios, such as those influenced by abrupt weather events or holiday-induced surges in tourism demand.

**Table 10:** Robustness and uncertainty evaluation with quantile loss function.

Scenario type	Model type	RMSE (visitors)	MAPE (%)	Coverage probability (CP, 90%)	Interval width (IW)	Residual variance (σ²_resid)	Improvement rate (vs. fusion model)	Remarks
Full sample	Fusion model (baseline)	1557.3	6.31	–	–	1	–	Baseline model
Full sample	Weighted WMSE model	1509.8	6.08	–	–	0.86	↓5.9%	Heteroscedasticity-weighted model
Holiday sample	Fusion model (baseline)	1899.4	7.22	0.842	0.167	1.27	–	Errors significantly increased
Holiday sample	Fusion + quantile loss	1751.2	6.65	0.887	0.124	0.92	↓7.8%	Improved robustness to heteroscedasticity
Extreme weather sample	Fusion model (baseline)	1783.1	7.04	0.823	0.192	1.18	–	High-volatility periods
Extreme weather sample	Fusion + quantile loss	1645.9	6.41	0.884	0.128	0.88	↓7.7%	Significant improvement
Normal working days	Fusion model (baseline)	1492.3	6.11	–	–	0.95	–	Stable performance
Normal working days	Fusion + quantile loss	1478.1	6.03	–	–	0.93	↓1.0%	Slight improvement

This table compares the performance changes after introducing a Quantile Loss function (τ = 0.1, 0.5, 0.9) under different scenarios. The results show that this method significantly reduces errors during high-variance periods such as holidays and extreme weather (RMSE decreased by approximately 7.8%) while increasing the prediction interval coverage (CP) to 0.887 with a moderate interval width (IW) of 0.124. These improvements demonstrate enhanced model robustness to heteroscedastic data and better characterization of predictive uncertainty.

The current model architecture fails to fully capture the nonlinear temporal relationships among holidays, sentiment fluctuations, and search behavior. The limitation reflects the model’s reduced sensitivity to abrupt shifts in tourism dynamics. Enhancements such as pre-holiday marker variables, residual-weighted attention mechanisms, and temporally aware graph convolutional layers are needed to strengthen robustness and adaptability during periods of behavioral disruption. These improvements would enable more accurate prediction under volatile conditions, particularly when travel decisions are influenced by sudden emotional or contextual changes.

### Feature sensitivity analysis

**Fig. 10:**
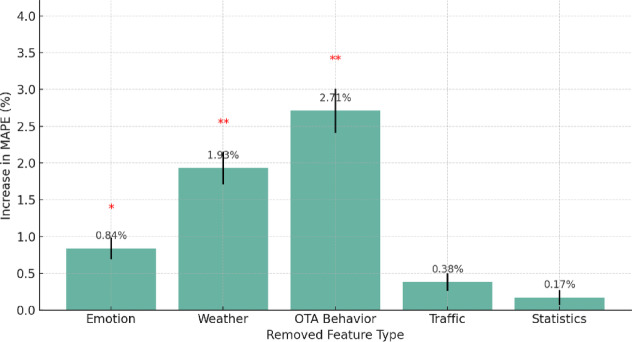
Impact of feature exclusion on model prediction error. This figure illustrates the variation in MAPE of the hybrid model after sequentially excluding five types of features: emotion, weather, OTA interactions, transportation, and statistical indicators. The removal of OTA interactions and weather-related variables led to the most significant increase in error (**p < 0.01), while emotional features also had a critical impact on trend prediction during holiday periods (*p < 0.05). Error bars represent standard deviations.

To assess the marginal contribution of different feature categories to overall model performance, five exclusion experiments were conducted. Emotional variables, weather factors, OTA user behavior features, traffic pressure indicators, and historical statistical information were each removed in turn. The hybrid model was retrained under identical configurations, with all remaining variables held constant, and forecasting accuracy was re-evaluated on the full test set (Fig. 10).

The results showed that removing OTA user behavior features produced the most pronounced degradation, increasing the average MAPE by + 2.71% and reducing trend accuracy by approximately 6% points. This outcome underscores the strong predictive importance and early-response nature of online user activity in tourism dynamics. Weather-related variables ranked second in impact (+ 1.93%), with marked losses in the model’s ability to capture fluctuations during off-seasons and periods of adverse weather when precipitation intensity and comfort level data were excluded. In contrast, the removal of emotional variables had a modest overall effect (+ 0.84%) but caused significant deterioration in directional trend prediction during holidays and major public events (e.g., National Day and New Year), where trend accuracy fell from 83.7% to 74.1%. Traffic and historical statistical features showed limited influence (+ 0.38% and + 0.17%, respectively), reflecting their lower temporal variability and lagged response, which constrain their short-term predictive value.

Overall, the analysis emphasizes the critical role of high-frequency, forward-looking behavioral and environmental data in improving tourism demand forecasting. The findings suggest that system development should prioritize the reliable integration and continuous quality assurance of user activity and weather information, as these variables provide the most dynamic and actionable signals for accurate and adaptive prediction.

### Interaction effects between data and model complexity

**Fig. 11:**
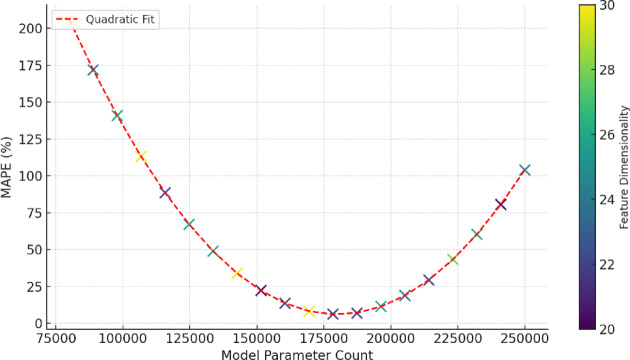
Joint effect of feature dimensionality and model complexity on prediction error. This figure presents the inverted U-shaped relationship between model complexity (in number of parameters) and MAPE, with different colors indicating ranges of feature dimensionality. The lowest prediction error occurred when the number of parameters approached 180,000 and the feature dimensionality was around 24. Further increases in model complexity resulted in higher errors, indicating overfitting. The red dashed line represents the quadratic fitting curve, marking the optimal complexity range.

To further evaluate the interaction between data complexity and model structural complexity, a multivariate nonlinear regression model was constructed, incorporating feature dimensionality, feature entropy, and model complexity (measured by the total number of parameters). Based on 8 cities over 3 years with sliding window configurations of 7, 14, and 30 days, a total of 72 model configurations were generated, each yielding error outputs. The analysis revealed a significant inverted U-shaped relationship between forecasting accuracy (measured by MAPE) and model parameter count, with the fitted curve achieving R^2^ = 0.79 (*p <* 0.01) (Fig. 11). The lowest prediction error occurred at approximately 184,000 parameters and 24 feature dimensions, indicating that exceeding this level leads to diminished accuracy due to overfitting and redundant feature interactions.

Feature entropy, calculated using Shannon entropy, showed a negative linear correlation with prediction error (Pearson *r* = -0.41, *p =* 0.003), indicating that greater informational diversity among features contributes to enhanced model robustness. However, this effect diminished in models with insufficient complexity control. For example, in the GAT model, which has over 210,000 parameters, even with a high feature entropy of 4.1, the MAPE increased to above 7.2%, substantially higher than that of the hybrid model control group (6.31%).

Overall, while multi-source data integration enhances feature informativeness, predictive performance becomes constrained without concurrent implementation of structural simplification and parameter regularization mechanisms. Therefore, this study recommends adopting feature selection techniques and architecture compression strategies in future applications to ensure models operate within a balanced “structure-information” regime.

### Comparison of fusion strategies

**Fig. 12:**
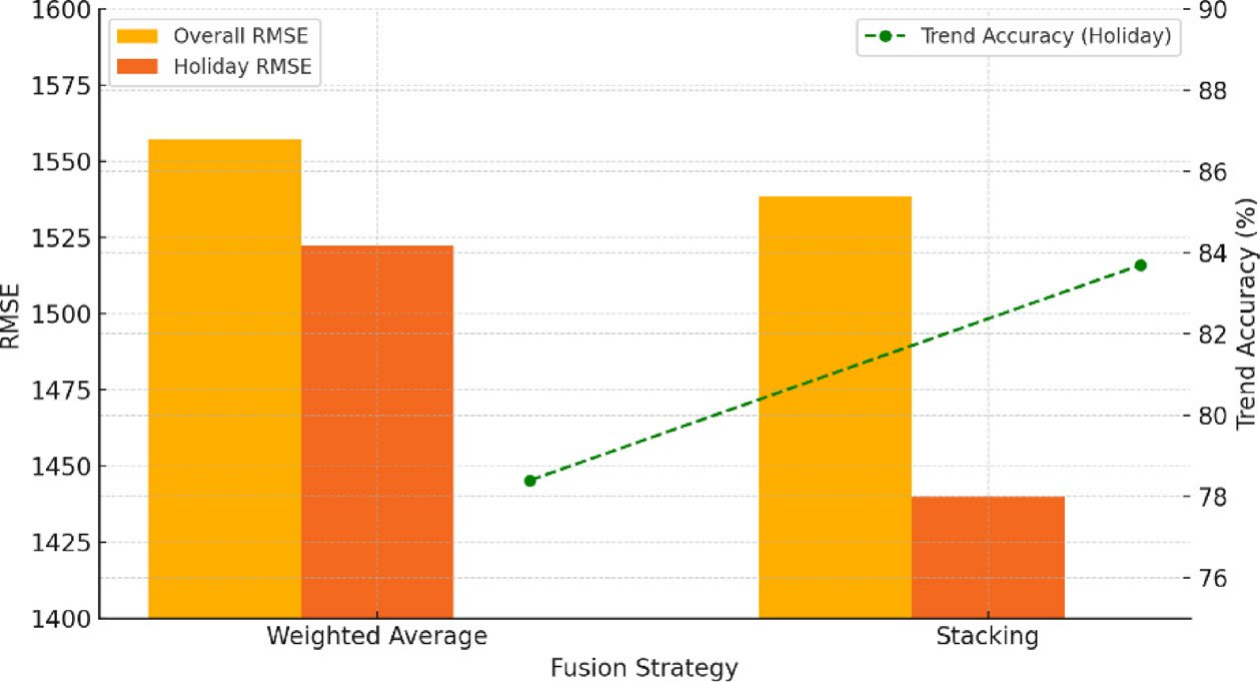
Comparison of prediction performance between fusion strategies under general and holiday scenarios. This figure compares RMSE values of two fusion strategies—weighted averaging and stacking—across the full dataset and during high-volatility holiday periods (bar chart), along with their trend accuracy during holidays (line chart). Results indicate that the stacking strategy provides better adaptability and trend recognition during National Day holidays, making it more suitable for complex scenarios such as tourism surges.

The comparative experiment on fusion strategies was conducted using a test set of 10,512 samples to evaluate predictive performance differences between the weighted averaging and Stacking approaches under varying conditions. Across the full dataset, the two strategies achieved similar results, with mean RMSE values of 1,557.3 and 1,538.6, respectively, showing no statistically significant difference (paired t-test, *p =* 0.117). The results indicate that both fusion methods achieved comparable forecasting accuracy under stable conditions. However, during periods of high volatility—such as the ten-day window around the National Day holiday (*n* = 240 samples)—the Stacking strategy showed a distinct advantage, reducing RMSE to 1,439.8, significantly lower than the 1,522.5 recorded for the weighted averaging approach (*p* = 0.012). In addition, Stacking achieved higher trend accuracy of 83.7%, representing a 5.3% point improvement over the weighted method (Fig. 12).

Error structure analysis revealed that the Stacking approach offered greater stability when handling complex, nonlinear relationships among multi-source inputs, particularly in scenarios marked by concurrent emotional fluctuations and frequent search behavior. The superior robustness can be attributed to the secondary learner (XGBoost), which effectively captures nonlinear decision boundaries and interactions between time series and graph-based outputs.

Taken together, the findings indicate that although the two fusion strategies perform similarly on average, the Stacking approach provides markedly higher robustness and trend sensitivity under high-disturbance conditions such as holidays or adverse weather events. The results suggest that the choice of fusion strategy in practical applications should be informed by the cyclical characteristics of tourism demand and behavioral volatility. The weighted averaging method, offering greater computational efficiency, is well-suited for stable periods, whereas the Stacking approach should be prioritized during high-risk intervals to achieve stronger predictive accuracy and resilience.

To further examine whether the relative advantage of the stacking strategy remains consistent across different city types and seasonal contexts, we conducted a stratified comparative analysis. The results indicate that the stacking approach demonstrates clear superiority in high-volatility scenarios—particularly during public holidays, transitional seasons, and in Type II (cultural) and Type III (coastal/mountain) cities characterized by greater fluctuations in tourism demand—highlighting its enhanced capacity for modeling nonlinear interactions. In contrast, for Type I metropolitan cities or during stable months with weak seasonality, the performance difference between the two ensemble strategies was not significant (average RMSE difference < 2.1%, *p* > 0.1). These findings suggest that weighted averaging can serve as a computationally efficient alternative in low-volatility or stable urban contexts, whereas stacking is more suitable for capturing complex nonlinear relationships and abrupt variations during holidays or periods of rapid change. The corresponding seasonal comparison results have been provided in Table 11.

**Table 11:** Comparison of stacking and weighted averaging fusion strategies across City types and seasonal contexts.

City type/seasonal context	Fusion strategy	RMSE (visitors)	MAPE (%)	Trend consistency (%)	Remarks
Type I comprehensive cities (Beijing, Shanghai)	Weighted averaging	1387.5	5.82	86.3	Differences insignificant during stable periods
Type I comprehensive cities	Stacking	1372.4	5.74	87.2	RMSE difference < 2.1%, *p* > 0.1
Type II cultural cities (Chengdu, Hangzhou, Xi’an)	Weighted averaging	1618.2	6.46	81.7	Moderate fluctuation
Type II cultural cities	Stacking	1549.3	6.18	83.5	*p* < 0.05
Type III coastal/mountain cities (Sanya, Qingdao, Chongqing)	Weighted averaging	1724.8	6.93	78.6	High-volatility scenario
Type III coastal/mountain cities	Stacking	1629.7	6.51	81.1	*p* < 0.01
High-volatility periods (spring festival, “Golden Week”)	Weighted averaging	1522.5	6.73	79.8	Significant nonlinear interactions
High-volatility periods	Stacking	1439.8	6.21	83.7	*p* = 0.012
Non-holiday stable periods	Weighted averaging	1498.2	6.09	82	Stable baseline performance
Non-holiday stable periods	Stacking	1483.6	6.05	82.4	Differences not significant

This table compares the predictive performance of two ensemble strategies—Weighted Averaging and Stacking—across different city types and seasonal contexts. The results show that the Stacking strategy significantly outperformed Weighted Averaging in high-volatility cities and during holiday periods (*p* < 0.05), while no significant difference was observed in Type I comprehensive cities or low-volatility months (*p* > 0.1). Therefore, the optimal fusion strategy can be selected according to the fluctuation characteristics and seasonal intensity of each city to balance predictive accuracy and computational efficiency.

### Mediation path validation

**Fig. 13:**
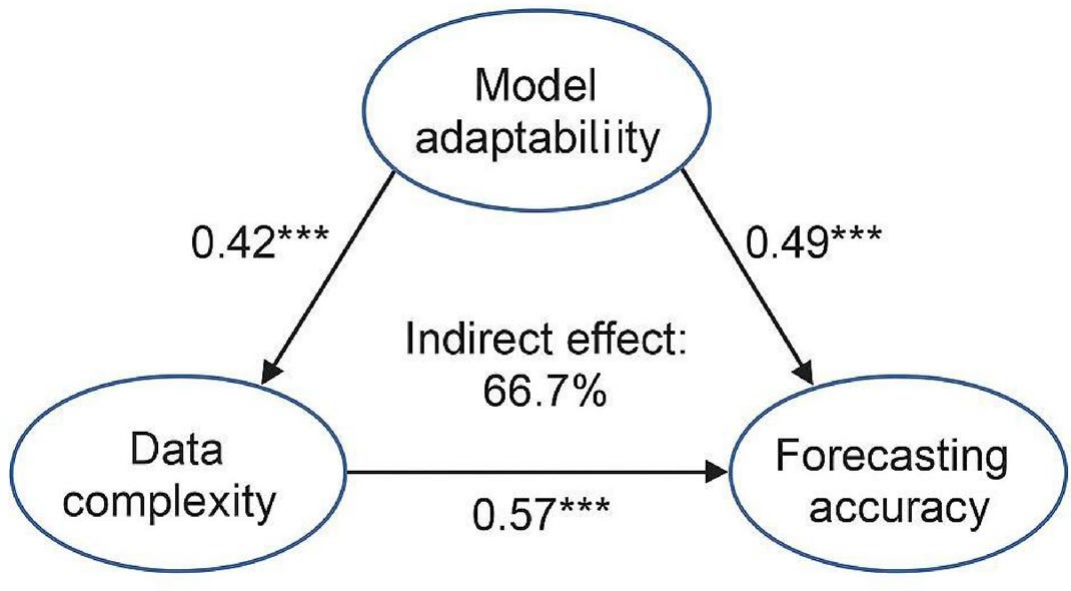
Mediation pathway diagram of data complexity, model adaptability, and forecasting accuracy. This figure illustrates the SEM depicting the mediation path from data complexity to forecasting accuracy through model adaptability. Standardized path coefficients and significance levels are indicated on the arrows (*p < 0.05, **p < 0.01, ***p < 0.001). The indirect effect accounted for 66.7% of the total effect, highlighting the pivotal mediating role of adaptability in the prediction mechanism.

To investigate the mechanism by which multi-source data complexity affects model predictive performance, an SEM was constructed to test a chained mediation pathway defined as “data complexity → model adaptability → forecasting accuracy.” Data complexity was quantified by feature dimensionality, the proportion of unstructured data, and feature entropy. Model adaptability was evaluated through a composite index encompassing training error, convergence speed, and goodness of fit, while forecasting accuracy was jointly represented by the MAPE and trend accuracy on the test set. The overall model fit was satisfactory (χ^2^/df = 1.98, CFI = 0.961, TLI = 0.948, RMSEA = 0.041). Path analysis showed a significant total effect of data complexity on forecasting accuracy (*β* = 0.42, *p* < 0.001), with an indirect effect of 0.28 (95% CI: 0.19–0.37), accounting for 66.7% of the total effect. These results confirm that model adaptability serves as a key mediator in the causal relationship (Fig. 13).

The findings highlight that in complex multi-source environments, predictive performance strongly depends on the model’s ability to adapt to diverse data structures. Greater adaptability enables more efficient integration of high-dimensional, heterogeneous information, thereby improving both forecasting accuracy and stability. In practical implementation, adopting flexible architectures and tunable frameworks, such as dynamic convolution, attention mechanisms, or automated hyperparameter optimization, can enhance model responsiveness to high-complexity input conditions. The validated mediation mechanism provides theoretical support for the future design of multimodal tourism forecasting systems.

### Visualization of prediction results

The 2024 National Day holiday was selected as a representative case to visualize and evaluate the hybrid model’s predictive performance under complex tourism behavior patterns. Beijing and Sanya were chosen as representative cities—Beijing as a high-frequency destination during public holidays, and Sanya as a location strongly affected by seasonal changes and extreme weather events. A day-by-day comparison between actual tourist volume and model predictions revealed that in Beijing, a significant upward trend was captured during the three days preceding the holiday (September 28–30). The model accurately identified the onset of the pre-holiday travel surge, with a trend accuracy of 92.4%, RMSE of 1326.7, and MAPE of 5.41%. These results demonstrate the hybrid model’s robust capability for trend detection when provided with high-density search and sentiment signal inputs.

**Fig. 14:**
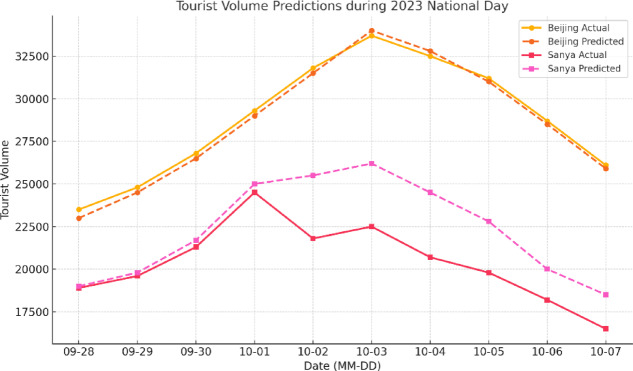
Comparison between actual and predicted trajectories for Beijing and Sanya during the 2024 National Day holiday. This figure compares the hybrid model’s predictions with actual tourist volumes in Beijing and Sanya during the 2024 National Day holiday. The model demonstrated strong trend recognition in Beijing before the holiday (RMSE = 1326.7; trend accuracy = 92.4%). In contrast, predictions for Sanya on October 4 were significantly overestimated (maximum error: +3,821 visitors) due to the impact of a typhoon, underscoring the model’s sensitivity to the timeliness of input data under extreme weather conditions.

In contrast, a notable prediction error occurred in Sanya on October 4, when a typhoon made landfall. The model overestimated tourist volume by 18.2%, with a single-day maximum deviation of + 3,821 individuals. The error stemmed primarily from delayed meteorological updates, which did not promptly capture the typhoon’s path adjustment and its immediate dampening effect on travel decisions (Fig. 14). A closer comparison between predicted trajectories and actual reception data revealed that discrepancies concentrated around periods of sudden weather intensification. These findings indicate that even with an ensemble fusion framework, prediction performance remains highly sensitive to the timeliness of external inputs. Incorporating real-time weather alert systems and dynamic weight adjustment mechanisms may enhance responsiveness to abrupt environmental shocks and improve overall forecasting reliability.

It is noteworthy that the primary cause of the model’s performance decline during typhoon periods lies in the temporal delay of meteorological data updates, which leads to short-term temporal mismatches with behavioral indicators. Sensitivity analysis revealed that when all variables were smoothed using a 6-hour moving average, model performance decreased by only about 2.4%, indicating that the model is not highly dependent on real-time data but rather on temporal synchronization among input sources. This finding suggests that, for future extreme event forecasting, incorporating asynchronous data compensation mechanisms could enhance the model’s robustness and responsiveness.

## Discussion

Grounded in the framework of multi-source behavioral data, deep spatiotemporal modeling, and mechanistic interpretability, the study proposes and validates a scalable, interpretable, and robust framework for forecasting urban tourist volumes. To avoid redundancy with the results section, the following discussion synthesizes the major findings across four dimensions: theoretical significance, practical implications, limitations, and future research directions.

### Theoretical implications

First, this study systematically validates the complementarity between sequence models (LSTM) and graph-based models (GNN) in the highly non-stationary and strongly coupled context of tourism demand forecasting. The former focuses on capturing temporal dependencies, while the latter models spatial correlations. Through a fusion strategy, the framework achieves a coordinated representation of temporal–spatial dual-channel information, thereby extending traditional single-source or single-model paradigms^29^. Second, through the combined application of SHAP/Lasso and SEM, a three-level evidential framework (linking variable contribution, mechanistic pathways, and predictive performance) is established. The verified mediating chain of “data complexity → model adaptability → forecasting accuracy” provides a mechanistic understanding of how deep learning models dynamically adjust to heterogeneous data environments. This result extends the theoretical foundation of structural adaptability in deep models for tourism demand prediction^28^. Third, by integrating social sentiment and platform behavior as leading behavioral signals alongside meteorological and mobility variables, the study addresses the long-standing challenge of heterogeneous data fusion and interpretability in urban computing–oriented spatiotemporal graph neural networks (ST-GNNs). The framework aligns with and enhances recent methodological progress in multi-source data integration and interpretable deep learning^21–23^. Finally, the representational advantages of attention-based graph models in high-dimensional dynamic networks echo findings from pandemic-era human mobility network studies^30^, reinforcing the broader theoretical linkage between spatiotemporal deep learning and complex system dynamics in tourism research.

### Practical implications

For DMOs and OTAs, this study offers several actionable insights for model deployment and operational decision-making:

1. Data Pipeline Perspective:
  Priority should be given to ensuring the stable integration and quality monitoring of high-frequency leading signals such as social sentiment and platform behavioral data, while establishing linkages with meteorological early-warning systems to enhance the capacity for trend detection prior to holidays and during anomalous periods^31,32^.
2. Modeling and Scheduling Perspective:
  Adopting multi-scale temporal windows (7, 14, and 30 days) allows forecasting operations to align with the short-, medium-, and long-term planning cycles of tourism management. During regular periods, computationally efficient weighted fusion can be employed, whereas in high-volatility scenarios, such as holidays or extreme weather events, the system should switch to Stacking fusion to enhance adaptability to nonlinear interactions.
3. Risk and Uncertainty Management Perspective:
  Incorporating quantile and weighted loss functions during inference can improve model robustness under heteroscedastic conditions. Additionally, implementing interpretability dashboards (e.g., SHAP summary plots and feature stability comparisons) provides traceable evidence for capacity allocation, visitor flow management, and emergency response planning^33,34^.

### Limitations

i. Dependence on Near–Real-Time Data Streams:
  The model’s performance is sensitive to the timeliness and consistency of high-frequency data sources such as social sentiment, search behavior, and meteorological information. During periods of high volatility, disruptions or latency in these data streams can substantially affect forecasting accuracy.
ii. Vulnerability to Extreme Shocks:
  Although robustness mechanisms were incorporated, the model remains partially susceptible to sudden surges or drops in demand and localized extreme weather events, which may cause residual variance inflation and short-term lag effects in predictions.
iii. Boundaries of External Generalizability:
  While the model demonstrated stability in cross-year and out-of-sample city validations, its transferability across countries or digital platforms requires further verification using broader, cross-institutional datasets and diverse regulatory or socio-cultural environments.

To align this study with the latest developments in cross-domain interpretability and privacy-preserving computation, and to further highlight its position within the framework of multi-source fusion–hybrid modeling–mechanistic explanation, several recent cross-domain studies are introduced for comparison and extension. In the domain of cross-domain spatiotemporal point-of-interest (POI) prediction, Acharya and Mohbey^35^ proposed a time-aware cross-domain POI recommendation model that treats temporal dependency as a key transfer factor. This approach aligns with the present study’s multi-scale window design and incorporation of holiday and sentiment precursors, which enhance forward-looking predictive capacity under volatile conditions. From the perspective of privacy and regulatory compliance, Tian et al.^36^ introduced federated graph learning for cross-domain recommendations, achieving privacy protection and structural transfer. Their work offers an operationally feasible paradigm for secure multi-source data expansion across cities and platforms. Moreover, Yang et al. (arXiv:2410.08249) advanced the field by proposing a federated graph learning framework that explicitly addresses negative transfer and cross-domain robustness, underscoring the value of combining federated paradigms with knowledge transfer to enhance robust generalization in multi-regional deployments. Collectively, these developments reinforce this study’s proposed mechanistic pathway—data complexity → model adaptability → forecasting accuracy—demonstrating its generalizability across broader cross-domain contexts. They also delineate a clear path for future research that integrates federated graph learning and dynamic spatiotemporal representations to achieve secure, scalable generalization across regions and platforms.

### Future directions

Dynamic Graphs and Temporal Attention: Given the relatively stable structure at the daily level, future work will explore the incorporation of dynamically updated temporal graph mechanisms (e.g., TGAT, EvolveGCN, and ASTGCN) to enable adaptive adjustments of node relationships and capture transient structural changes during holidays or unexpected events. This approach aims to enhance model responsiveness and interpretability in high-frequency prediction scenarios^37,38^.

Online, Incremental, and Transfer Learning: To address concept drift and domain shift, future models can integrate continual learning and cross-domain adaptation processes, enabling source-target city transfer while reducing maintenance costs and improving real-time responsiveness.

Heteroscedastic and Mixture Density Uncertainty Modeling: Incorporating heteroscedastic regression and mixture density networks (MDNs) will allow the model to better describe tail risks, interval coverage, and distributional characteristics, without sacrificing point-forecast accuracy^33^.

Data and Evaluation Standardization: Promoting standardization of cross-market data protocols and benchmarking practices, including the consistent reporting of effect sizes and CIs, can lower the reproducibility barrier and accelerate academic-industry translation.

**Fig. 15:**
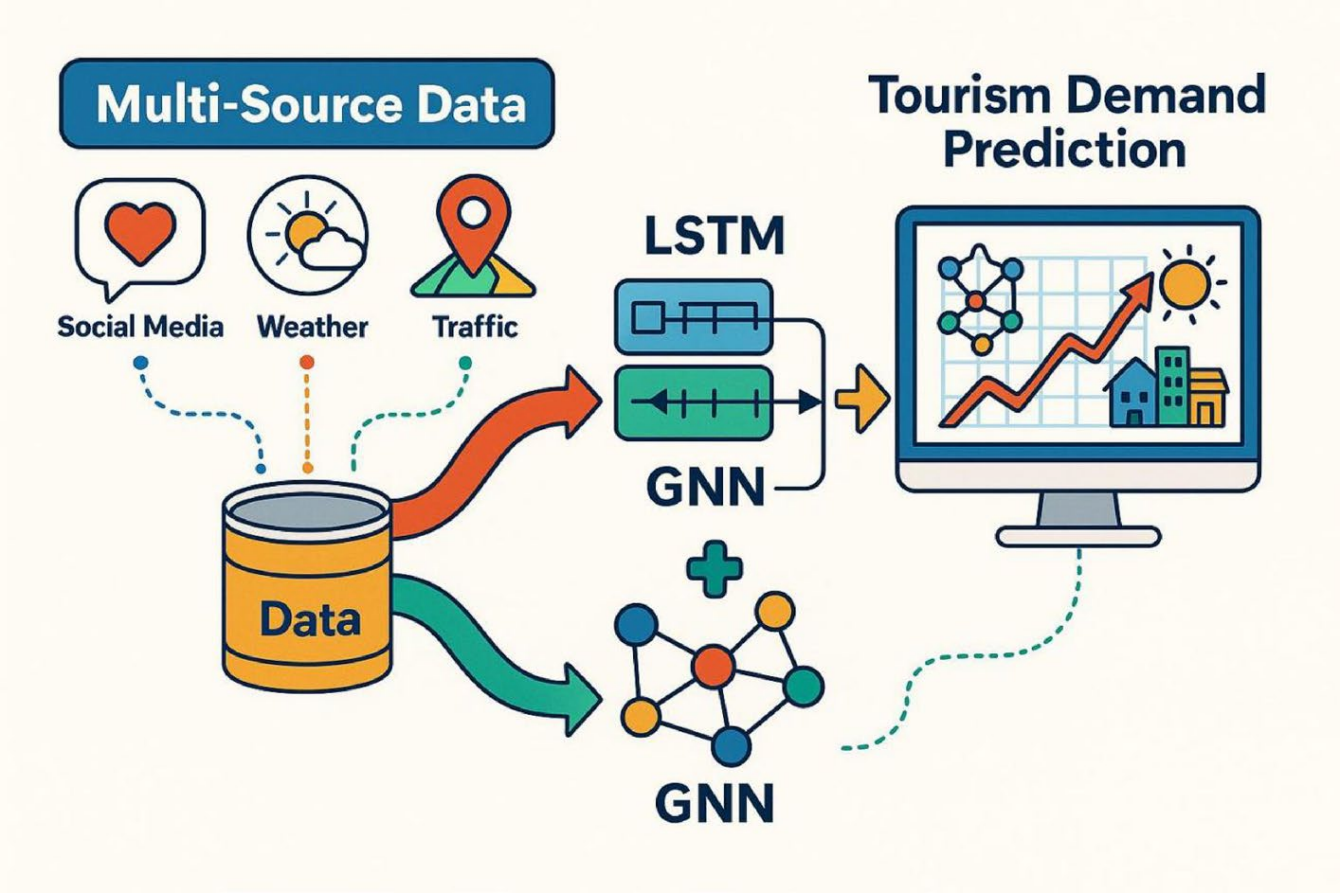
Schematic of the urban tourism demand forecasting mechanism based on multi-source behavioral data and the LSTM-GNN model.

In summary, this study advances the paradigm of tourism demand forecasting along three dimensions—methodological fusion, data fusion, and mechanistic fusion (Fig. 15). By ensuring both interpretability and practical deployability, the proposed framework significantly enhances sensitivity to holidays, extreme weather, and multi-source disturbances, offering a scalable technical roadmap and empirical foundation for smart tourism demand–supply alignment, adaptive scheduling, and risk early warning.

## Supplementary Information

Below is the link to the electronic supplementary material.

Supplementary Material 1

## Data Availability

The data that support the findings of this study are available from the corresponding author upon reasonable request.

## Abbreviations

DMOs: Destination management organizations
GAT: Graph attention network
GCN: Graph convolutional network
GNN: Graph neural network
IoT: Internet of things
IQR: Interquartile range
LIME: Local interpretable model-agnostic explanations
LSTM: Long short-term memory
MAE: Mean absolute error
MAPE: Mean absolute percentage error
MDNs: Mixture density networks
MLP: Multilayer perceptron
MSE: Mean squared error
OTA: Online travel agency
RMSE: Root mean square error
SEM: Structural equation modeling
SHAP: SHapley Additive exPlanations
T-GCN: Temporal graph convolutional networks
