# Supplementary material for: A multi-source behavioral data framework for interpretable urban tourism forecasting
Source: Sci Rep. 2025 Dec 24;16:2257. doi: 10.1038/s41598-025-32127-2 (PMC12816594; doi:10.1038/s41598-025-32127-2)
Supplement: Supplementary file 1 — Supplementary Material 1 [file 41598_2025_32127_MOESM1_ESM.docx]

**Appendix S1 — Details of Data Cleaning and Feature Engineering**

**Purpose:** This appendix provides the implementation details of the procedures described in the main text under “Data Cleaning and Harmonization” and “Feature Engineering and Data Fusion”, to ensure reproducibility and methodological transparency. The main article only summarizes the methodological rationale and selection principles.

**S1.1 Data Cleaning and Harmonization**

**Missing values and outliers:**

-Continuous variables were imputed via linear interpolation. Discrete and periodic variables (e.g., holidays, weather grades) were imputed using city × weekday mean values.

-Outliers were detected using a dual IQR and ±3σ criterion, with the removal rate controlled below 2% to maintain sample stability.

**Temporal alignment**

-All source data were **resampled to a daily frequency**. High-frequency event data (e.g., microblogs, transportation logs) were **mapped to observation days** to eliminate cross-day drift.

**Spatial standardization**

-A **city-coding dictionary** was built to standardize place names, attractions, and GPS data across the eight target cities.

**-Jaccard-based token matching** combined with **TF-IDF similarity** was used for semantic normalization and geolocation inference. Nonstandard toponyms (e.g., “West Lake”, “Tian’anmen Square”) in OTA and transportation data were refined through **NLP-based matching plus manual validation**, achieving >96% accuracy.

**City–attraction graph construction**

-A **city–attraction structural graph** was established under the 8-city framework. Each node was assigned its daily time-series features, and **edge weights** were defined as a weighted combination of tourist mobility intensity × geographic distance, serving as the input topology for the GNN model.

**Text and sentiment analysis pipeline**

-Text preprocessing: Chinese word segmentation via **jieba**, with stopword and noise filtering.

-Lexicon-based channel: Sentiment polarity was computed using the **BosonNLP Chinese Sentiment Lexicon**.

-Pre-trained model channel: A fine-tuned **Chinese BERT encoder** was coupled with a binary sentiment classifier to generate polarity scores in the range [-1, 1].

-Fusion: The two channels were integrated by performance-based weighting on the validation set to yield the **daily mean sentiment index** per city, from which sentiment volatility (standard deviation) and polarity bias (positive–negative ratio difference) were derived.

**S1.2 Feature Engineering and Data Fusion**

**Multi-scale temporal windows**

-Sliding windows of **7, 14, and 30 days** were used to capture short-term shocks, mid-term trends, and long-term inertia. For each city and each day, training samples were generated with prediction targets of **T+1 day** or **the following 7 days’ tourist volume**.

**Dataset size**

-Covering 2022–2024, a total of **70,080 samples** were generated (8 cities × 365 days × 3 years), by rolling through the window.

**Feature groups and definitions**

-Behavior: Mean and SD of sentiment indices; OTA search volume, review count, and booking conversion rate

-Environment: Temperature, precipitation, holiday indicator, and comfort index (1–5, based on meteorological standards)

-Mobility: Average and peak congestion index; traffic flow near attractions; count and density of signal-based gathering regions

-History: 7-, 14-, and 30-day lagged means of tourist volume

**Distribution stabilization and scaling**

-Numerical features underwent skewness testing and Box–Cox transformation, with λ estimated via maximum likelihood (MLE) on the training set.

-Behavioral intensity features were normalized via min–max scaling between the 1st–99th percentiles, mitigating the influence of outliers.

-All scaling parameters were frozen during validation and testing to prevent data leakage.

**Unified input tensor**

-A fused feature tensor with shape (T × 28) was constructed (T ∈ {7, 14, 30}), integrating both structured and unstructured information. This provided a consistent time–feature input interface for the LSTM, GNN, and hybrid fusion models.

**S1.3 Reproducibility Notes**

-All preprocessing and feature pipelines were identically applied across training, validation, and testing sets.

-Key thresholds (e.g., outlier filters and fusion weights) were determined via cross-validation within the training set.

-Full implementation code and parameter tables can be provided in accordance with the journal’s data-sharing policy.
